# Organogermanium Analogues of Alkenes, Alkynes, 1,3-Dienes, Allenes, and Vinylidenes

**DOI:** 10.3390/molecules28041558

**Published:** 2023-02-06

**Authors:** Vladimir Ya. Lee

**Affiliations:** Department of Chemistry, Faculty of Pure and Applied Sciences, University of Tsukuba, Tsukuba 305-8571, Ibaraki, Japan; leevya@chem.tsukuba.ac.jp

**Keywords:** bond length, double bond, germanium, main group element, multiple bonding, NMR spectroscopy, theoretical calculations, torsional angle, *trans*-bending, X-ray crystallography

## Abstract

In this review, the latest achievements in the field of multiply bonded organogermanium derivatives, mostly reported within the last two decades, are presented. The isolable Ge-containing analogues of alkenes, alkynes, 1,3-dienes, allenes, and vinylidenes are discussed, and for each class of unsaturated organogermanium compounds, the most representative examples are given. The synthetic approaches toward homonuclear multiply bonded combinations solely consisting of germanium atoms, and their heteronuclear variants containing germanium and other group 14 elements, both acyclic and cyclic, are discussed. The peculiar structural features and nonclassical bonding nature of the abovementioned compounds are discussed based on their spectroscopic and structural characteristics, in particular their crystallographic parameters (double bond length, *trans*-bending at the doubly bonded centers, and twisting about the double bond). The prospects for the practical use of the title compounds in synthetic and catalytic fields are also briefly discussed.

## 1. Introduction

One of the most fundamental topics in modern organogermanium chemistry is the study of low-coordinate species, and within this realm, the field of multiply bonded compounds is now one of the mainstreams. It therefore comes as no surprise that the literature covering the latter field is vast. In this review, we limit our discussion to unsaturated organogermanium compounds (i.e., heavier analogues of alkenes, alkynes, 1,3-dienes, allenes, and vinylidenes) reported in the literature, mostly from 2000 to the present date, considering only combinations between the group 14 elements of the types >Ge=E< and –Ge≡E– (E = group 14 element). Heteronuclear multiply bonded combinations of germanium with the Main Group elements of groups 13, 15, and 16, >Ge=E^13^–, >Ge=E^15^–, and >Ge=E^16^, respectively, are excluded from our consideration. Likewise, aromatic organogermanium compounds (such as germabenzene, germanaphthalene, etc.), as well as germylene and germylyne transition metal complexes with Ge=M and Ge≡M bonds (M = transition metal), are also outside of the scope of this review and are not discussed. Moreover, numerous compounds, in which the low-coordinate Ge center is *intra*molecularly (by *n*-donor substituents) or *inter*molecularly (through external donor ligands) coordinated, thus experiencing remarkable electronic perturbation, are also not considered, except for silagermenylidenes >Si=Ge(NHC): and digermanium(0) complexes :Ge^0^(NHC/or NHSi)=Ge^0^(NHC/or NHSi):, which otherwise cannot be stabilized for their isolation.

For each class of unsaturated organogermanium compounds, the discussion starts with a brief introduction of the first stable representatives going on to consider their most important examples reported from 2000 to date. For a more comprehensive reading on the topic of multiply bonded organogermanium compounds, we refer interested readers to the previously published reviews [1,2,3,4,5,6,7].

**Figure 1:**
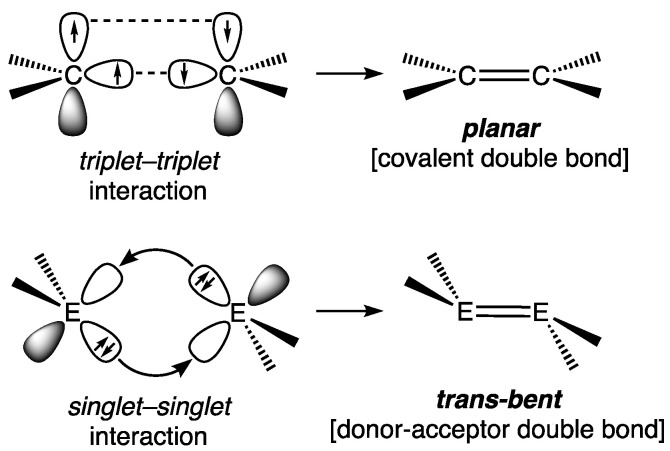
Formation of the planar alkene C=C by the triplet–triplet interaction of two carbenes >C: vs. formation of the *trans*-bent heavy alkene analogue E=E by the singlet–singlet interaction of two heavy carbene analogues >E:.

The peculiar structure and bonding nature of the doubly and triply bonded derivatives of the heavy group 14 elements that are distinctly different from those of their organic counterparts (that is, alkenes and alkynes) have been the subject of numerous comprehensive theoretical and experimental treatments described in several reviews [1,2,3,4,5,6,7]. Thus, in contrast to organic alkenes C=C and alkynes C≡C, which are typically planar and linear, respectively, their heavy tetrel analogues of the type E=E and E≡E (E = Si–Pb) are not planar and linear. Thus, for example, for the heavy alkene analogues E=E, there are two fundamental structural deformations leading to a departure from planarity: *trans*-bending at the doubly bonded E centers and twisting about the E=E bond. However, if twisting of the double bond generally results from the steric hindrance caused by the presence of exceptionally bulky substituents, the origin of the *trans*-bending and, thus, pyramidalization at E centers is best rationalized in terms of the MO interaction approach. Several qualitative bonding models have been proposed to account for the geometrical distortions in alkene analogues of the heavy group 14 elements. The first one, proposed by Lappert and coworkers, rationalized the *trans*-bending in E=E as a result of double donor-acceptor interaction of two singlet fragments [E↑↓], as opposed to the covalent interaction of the two triplet fragments [↑C↑], resulting in the formation of planar alkene C=C (Figure 1) [8].

**Figure 2:**
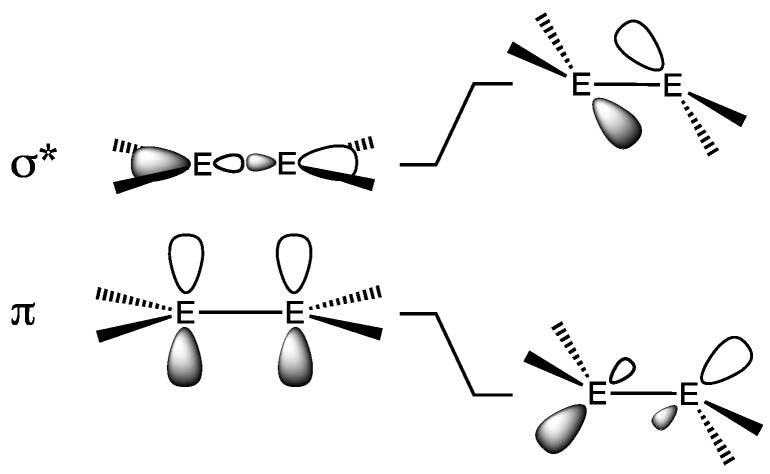
Second-order Jahn–Teller *π*–σ* orbital interaction accounting for the *trans*-bent distortion in the analogues of alkenes of the heavy group 14 elements.

Alternatively, the geometrical deviation from planarity (in alkenes) to *trans*-bent structures (in heavy alkene analogues) can be treated as a manifestation of a second-order Jahn–Teller effect in the form of the interaction of MOs having the same symmetry [1,4,5,9]. Specifically, this is mixing of HOMO(*π*_E=E_) and LUMO+1(σ*_E=E_) orbitals, stabilizing the HOMO and destabilizing the LUMO+1 and thus generating nonbonding electron density at E, which finally results in the observed *trans*-bending caused by the electronic repulsion (Figure 2). This *π*–σ* orbital interaction is facilitated for Ge-containing heavy alkene analogues, because the energy difference (inversely proportional to the extent of a second-order Jahn–Teller interaction) between the interacting orbitals is lower for the heavier tetrels due to their progressively decreasing double-bond strength.

Likewise, the *trans*-bent geometry of the heavy alkyne analogues E≡E (E = Si–Pb), as compared to the linear arrangement of organic alkynes C≡C, can also be realized in terms of the second-order Jahn–Teller MO interactions [3,4,5,9] (for the effect of the alkali metal counter ions on the structure of the digermyne doubly reduced salts, see [10]).

**Figure 3:**
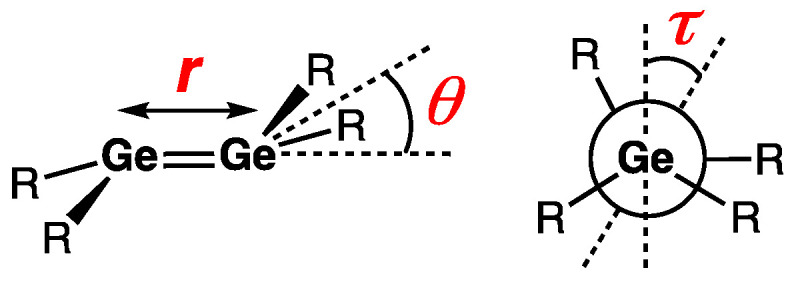
Structural parameters of digermenes (and other multiply bonded organogermanium compounds): bond length, ***r*Ge=Ge**; bent angle ***θ***; and twist angle ***τ***.

^73^Ge NMR spectroscopy has a very limited practical applicability for identification of organogermanium compounds, caused by the large quadrupole moment and low sensitivity of the ^73^Ge nucleus, resulting in a significant broadening of its resonance signals. Therefore, the paramount structural information on the multiply bonded organogermanium compounds can be obtained from their X-ray crystallography studies. Accordingly, the following structural parameters are most essential for the discussion of the particular bonding situations in the multiply bonded organogermanium compounds (as a most representative example, digermenes >Ge=Ge< are depicted below): (a) Ge=Ge bond length, ***r*_Ge=Ge_**(in Å) [typically, short]; (b) Ge=Ge bond substituents arrangement, as defined by either bent angle, ***θ*** (in °) [typically, *trans*-bent (see above)], or the sum of the bond angles around the sp^2^-Ge atoms, ***Σ*_Ge_** (in °); and (c) Ge=Ge bond twisting, ***τ*** (in °) [typically, not twisted] (Figure 3). Below, the bonding situation in unsaturated organogermanium compounds will be discussed based on these structural parameters (***r*_Ge=Ge_**, ***θ***, ***Σ*_Ge_**, and ***τ***).

## 2. Heavy Analogues of Alkenes

### 2.1. Homonuclear Derivatives

#### 2.1.1. Digermenes >Ge=Ge<

##### Acyclic Digermenes

**Scheme 1:**
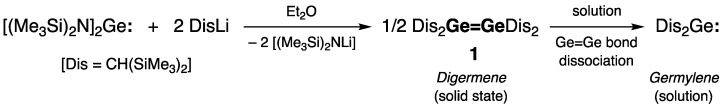
Synthesis of the first isolable digermene Dis2Ge=GeDis2 **1**.

The very first isolable digermene, namely tetra(alkyl)digermene Dis_2_Ge=GeDis_2_ [Dis = CH(SiMe_3_)_2_] **1**, reported by Lappert and coworkers in 1976, was synthesized by the reaction of bis(amino)germylene [(Me_3_Si)_2_N]_2_Ge: with DisLi [11], and its crystal structure was reported ten years later (Scheme 1) [12]. Dimeric was in the solid state (as was confirmed by X-ray diffraction study), and digermene **1** dissociated in solution into a pair of monomeric germylenes Dis_2_Ge:, thus implying the easy breaking of the weak Ge=Ge double bond (Scheme 1). Remarkably, crystallographic studies revealed that the Ge=Ge bond in **1** was notably short (*r*_Ge=Ge_ = 2.347(2) Å) and not twisted, the geometry around both Ge centers was pyramidal (***Σ***_Ge_ = 348.5°), and the Dis-substituents at the Ge=Ge double bond were arranged in a *trans*-bent fashion (*θ* = 32°).

**Scheme 2:**
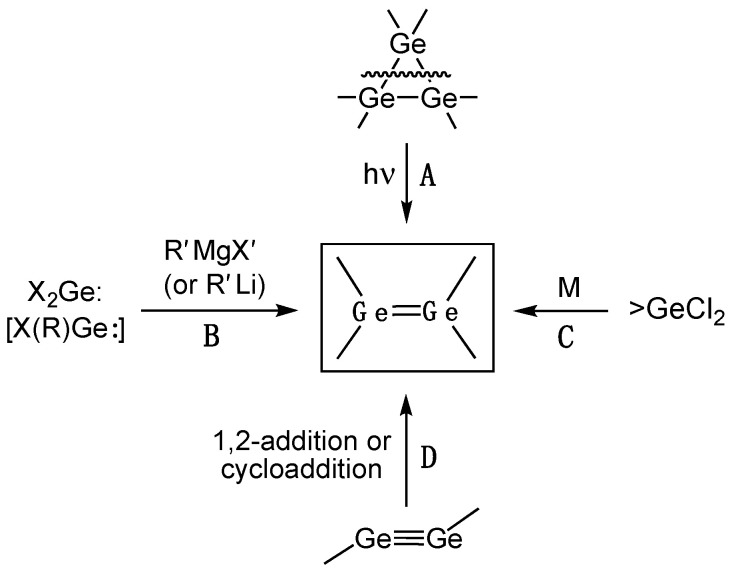
General methods for the synthesis of isolable digermenes.

Since 1976, numerous digermenes have been isolated and, in the majority of cases, structurally characterized, with most of these cases being reported after 2000. The synthetic strategies toward stable digermenes can be categorized into the four main approaches: (1) photolysis of cyclotrigermanes (route **A**); (2) reduction of dihalo- or monohalogermylenes with Grignard or organolithium reagents (route **B**); (3) reductive dehalogenation of 1,1-dihalogermanes (route **C**); and (4) 1,2-addition or cycloaddition to digermynes (route **D**) (Scheme 2).

Route **A** (photolysis of hexaarylcyclotrigermanes) is mostly of a historical importance as the method employed by Masamune and coworkers for preparation of the first stable tetra(aryl)digermenes Ar_2_Ge=GeAr_2_ [13]. However, because of the synthetic limitations of this approach, which requires cyclotrogermane precursors that are not readily available, currently this method is not commonly used. Since 2000, there was only one report from the Baines group on an improved synthetic procedure for the tetra(mesityl)digermene Mes_2_Ge=GeMes_2_ **2** that was generated by photolysis of hexa(mesityl)cyclotrigermane precursor in THF at −70 °C [14].

According to approach **B**, the reduction of isolable di(halo)germylenes X_2_Ge:/(X_2_Ge:–dioxane complex) or mono(halo)germylenes X(R)Ge: with Grignard RMgX or organolithium RLi reagents generates at first transient germylenes R_2_Ge: that dimerize forming digermenes R_2_Ge=GeR_2_, and this method was used for the preparation of Lappert’s germylene Dis_2_Ge=GeDis_2_ **1** [11,12]. Since 2000, a few other isolable digermenes were prepared by method **B**: Ar(R)Ge=Ge(Ar)R [R = Me, Et, Ph; Ar = 2,6-(2,4,6-*^i^*Pr_3_-C_6_H_2_)_2_-C_6_H_3_] **3a**–**c** [15], [Me_3_Si–C≡C](Ar′)Ge=Ge[C≡C–SiMe_3_]Ar′ [Ar′ = 2,6-(2,6-*^i^*Pr_2_-C_6_H_3_)_2_-C_6_H_3_] **4** [16], Ar*Ge=GeAr* [Ar* = 2,5-*^t^*Bu_2_-C_6_H_3_] **5** [17], Bbt(Br)Ge=Ge(Bbt)Br [Bbt = 2,6-[(Me_3_Si)_2_CH]_2_-4-[(Me_3_Si)_3_C]-C_6_H_2_] **6** (equilibrating in solution with the monomeric germylene Bbt(Br)Ge:) [18], and Tbb(Br)Ge=Ge(Tbb)Br [Tbb = 2,6-[(Me_3_Si)_2_CH]_2_-4-*^t^*Bu-C_6_H_2_] **7** [19]. A donor-acceptor Lewis base–Lewis acid digermene complex {[(OC)_5_W]←:GeH_2_–H_2_Ge←[:IPr]} [IPr = 1,3-bis(2,6-diisopropylphenyl)-2*H*-imidazol-2-ylidene] **8** was also prepared, featuring however single (instead of double) germanium–germanium bond, being therefore not classified as a true digermene [20].

Reductive dehalogenation of 1,1-di(halo)germanes (method **C**) is by far the most popular protocol for synthesis of stable digermenes, due to the ready availability of the starting R_2_GeX_2_. Using this approach, the synthesis and crystal structure of the following digermenes were reported: (R_3_Si)_2_Ge=Ge(SiR_3_)_2_ [R_3_Si = SiMe_2_*^t^*Bu] **9** [21,22], Tbt(Mes)Ge=Ge(Tbt)Mes [Tbt = 2,4,6-[(Me_3_Si)_2_CH]_3_-C_6_H_2_] **10** [23], Fc(Tip)Ge=Ge(Fc)Tip [Tip = 2,4,6-*^i^*Pr_3_-C_6_H_2_, Fc = ferrocenyl] **11** [20]. In tetra(aryl)digermene **10**, the strong repulsive interaction of the bulky aryl substituents caused the room-temperature dissociation of the Ge=Ge bond into the corresponding germylenes Tbt(Mes)Ge:, as was monitored by UV–Vis spectroscopy (Δ*H* = 14.7 ± 0.2 kcal/mol, Δ*S* = 42.4 ± 0.8 cal/mol·deg) [24].

Method **D**, namely 1,2-addition or cycloaddition across the Ge≡Ge triple bond of digermynes, is the latest approach toward digermenes that was enabled by the recent availability of the stable digermynes. This approach is exemplified by the preparation of Ar′(H)Ge=Ge(Ar′)H [Ar′ = 2,6-(2,6-*^i^*Pr_2_-C_6_H_3_)_2_-C_6_H_3_] **12** [25] (which is also available by method **B** [26]) and Ar′(H)Ge=Ge(Ar′)R [Ar′ = 2,6-(2,6-*^i^*Pr_2_-C_6_H_3_)_2_-C_6_H_3_, R = cyclopentyl] **13** [27]. Using method **D**, several compounds with a Ge–Ge bond were classified as digermenes [28,29,30]. However, given their remarkably long (even longer than many Ge–Ge single bonds) and accordingly quite weak Ge=Ge bonds, this classification is somewhat doubtful.

Below, some recently published representative examples of the stable digermenes are described.

Thus, employing method **C**, Lee, Sekiguchi and coworkers reported the tetra(silyl)digermene (*^t^*Bu_2_MeSi)_2_Ge=Ge(SiMe*^t^*Bu_2_)_2_ **14**, featuring very bulky substituents, that was readily available in large-scale by the reductive dehalogenation of the (*^t^*Bu_2_MeSi)_2_GeCl_2_ precursor with potassium graphite [31,32,33]. In the solid state, digermene **14** manifested a quite unusual combination of the structural features, namely a very long [*r*_Ge=Ge_ = 2.346(2) Å] and exceptionally twisted (*τ* = 52.8°) Ge=Ge double bond, nevertheless featuring practically planar geometry at its sp^2^-Ge centers (***Σ***_Ge_ = 358.8 and 359.2°) [31,32]. In solution, digermene **14** has a very distinct deep sapphire-blue color, in contrast to all other isolable digermenes that are yellow, orange, or red. Accordingly, its longest wavelength UV band was observed at 618 nm [*π*(HOMO)–π*(LUMO)], a value that was extraordinarily red-shifted compared to those of other stable digermenes [31,32]. This was caused by the extreme twisting of the Ge=Ge double bond due to the severe steric repulsion of bulky silyl substituents, resulting in rather poor 4*p*_π_(Ge)–4*p*_π_(Ge) orbital overlap, destabilization of the HOMO, and accordingly to the overall decrease in the HOMO–LUMO energy gap. Moreover, exceptional twisting of the Ge=Ge bond in **14** results in its partial breaking and progressively increasing biradical contribution. Digermene **14** does not dissociate in solution into the germylenes (*^t^*Bu_2_MeSi)_2_Ge:, maintaining its structural integrity up to 80 °C, as confirmed by Raman and UV–Vis spectroscopy measurements, as well as trapping reactions study [31,32,33]. Nevertheless, **14** can behave as a germylene source when strongly nucleophilic Lewis bases (isocyanide or *ortho*-benzoquinone) are applied, forming germylene reactivity products although not involving “free” germylenes into the reaction process [31,32]. CV measurement of digermene **14** in *ortho*-dichlorobenzene in the presence of [*^n^*Bu_4_N][B(C_6_F_5_)_4_] inert electrolyte afforded two reversible redox couples, for both oxidation and reduction processes, with *E*_1/2_(ox) = 0.38 V and *E*_1/2_(red) = −1.50 V [32]. This implies that both cation-radical [14]^+∙^ and anion-radical [14]^−∙^ are persistent under the CV measurement conditions.

**Scheme 3:**
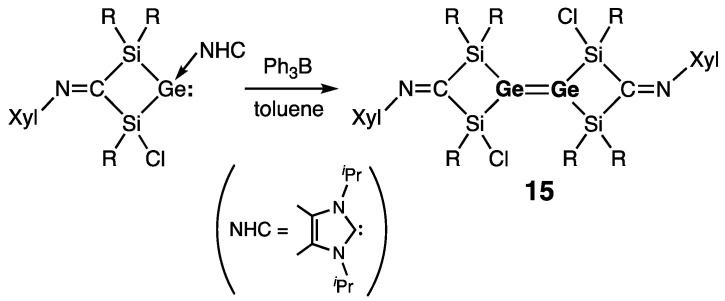
Synthesis of the tetra(silyl)digermene **15**.

Scheschkewitz and coworkers synthesized tetra(silyl)digermene **15** by dimerization of a cyclic germylene–NHC complex (Scheme 3) [34]. The exocyclic Ge=Ge bond in **15** revealed structural features that are typical for digermenes: short bond [*r*_Ge=Ge_ = 2.2944(4) Å], strongly pyramidalized Ge centers [***Σ***_Ge_ = 334.5°], and *trans*-bending of the silyl-substituents [*θ* = 37.7°]. Accordingly, digermene **15** was stable both in the solid state and in solution.

The same group also recently reported the first isolable digermenide Tip_2_Ge=Ge(Tip)Li **16** made by the reduction of Tip_2_GeCl_2_ with metallic lithium [35]. In this anionic digermene, the Ge=Ge double bond is short [*r*_Ge=Ge_ = 2.284(6) Å], unremarkably *trans*-bent (*θ* = 7.1/12.8°), and twisted (*τ* = 19.9°). Digermenide **16** can be further functionalized at the anionic Ge site forming novel silyl-substituted digermenes Tip_2_Ge=Ge(Tip)R (R = SiMe_3_ [35], SiPhMe_2_ [35], SiPh_3_ [36], SiMe_2_Cl [36], SiMePhCl [36], and SiPh_2_Cl [36]) and even persistent (acyl)digermenes Tip_2_Ge=Ge(Tip)[C(O)R] (R = *^t^*Bu [36], 2-methylbutan-2-yl [36], 1-adamantyl [36]).

Matsuo, Sasamori and coworkers prepared 1,2-di(halo)digermenes Eind(X)Ge=Ge(X)Eind (X = Cl, Br) **17a,b** (a: X = Cl, b: X = Br) by the redistribution of stable di(aryl)germylene Eind_2_Ge: and GeX_2_∙dioxane complex [37]. Bearing electronegative substituents, **17a,b** expectedly revealed strong structural deformation at the Ge=Ge double bond: *trans*-bending of substituents (*θ* = 44.3/43.3°) and pyramidalization at the Ge centers (***Σ***_Ge_ = 335.9 and 337.1°). In line with this, the Ge=Ge bond in **17a**,**b** is rather long [*r*_Ge=Ge_ = 2.4119(5) and 2.4145(3) Å], as a manifestation of the weak bonding between Ge atoms, which was translated into the ready dissociation of this bond. Accordingly, in solution, di(halo)digermenes Eind(X)Ge=Ge(X)Eind dissociate to (halo)germylenes Eind(X)Ge:.

**Scheme 4:**
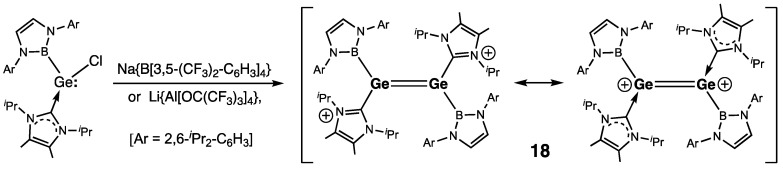
Synthesis of the dicationic digermene **18**.

Dicationic derivative **18**, which can be viewed as a digermene with cationic imidazolium substituents, as reported by Aldridge and coworkers, was synthesized by the reaction of a (chloro)germylene–NHC complex with either Na{B[3,5-(CF_3_)_2_-C_6_H_3_]_4_} or Li{Al[OC(CF_3_)_3_]_4_} (Scheme 4) [38]. In **18**, the Ge=Ge bond is short [*r*_Ge=Ge_ = 2.300(2) Å], and Ge centers are only insignificantly pyramidalized (***Σ***_Ge_ = 353.1 and 353.6°). Exchanging in **18** NHC substituents for Me_4_-NHC and boryl substituents for Ar-groups [Ar = 2,6-Mes_2_-C_6_H_3_ (Mes = 2,4,6-Me_3_-C_6_H_2_)], the same group synthesized another dicationic digermene **19** manifesting longer Ge=Ge distance of 2.380(1) Å [39].

Very recently, Scheschkewitz and coworkers found that the thermolysis (benzene, 65 °C, 18 h) of the unsymmetrically substituted digermene Tip_2_Ge=Ge(Tip)[SiR_2_Dma] (R = Me, Ph; Dma = 2-Me_2_N-C_6_H_4_) produced a mixture of redistribution products, Tip_2_Ge=GeTip_2_ and *trans*-[DmaR_2_Si](Tip)Ge=Ge(SiR_2_Dma)Tip **20** [Ge=Ge bond (for R = Me): *r*_Ge=Ge_ = 2.2576(5) Å, *θ* = 21.5°, *τ* = 0°] [40]. This approach was then applied toward the development of ADMET (acyclic diene metathesis) polymerization of digermenes. Accordingly, thermolysis (benzene, 65 °C, 48 h) of a diene with terminal digermene fragments linked by a *p*-phenylene spacer, Tip_2_Ge=Ge(Tip)–SiMe_2_–1,4-[2,5-(Me_2_N)_2_-C_6_H_2_]–SiMe_2_–Ge(Tip)=GeTip_2_ **21** [Ge=Ge bonds: *r*_Ge=Ge_ = 2.3038(4) Å, *θ* = 24.9/31.9°, *τ* = 18.0°], formed ADMET-polyene Tip_2_Ge{=Ge(Tip)–SiMe_2_–1,4-[2,5-(Me_2_N)_2_-C_6_H_2_]–SiMe_2_–Ge(Tip)=}*_n_*GeTip_2_, with the number-average degree of polymerization being 23, mass-average degree of polymerization being 45, and dispersity index being 1.95.

In comparison to organic alkenes >C=C<, structural deformations of the double bond in digermenes >Ge=Ge< (stretching, *trans*-bending, and twisting) are even more pronounced compared to those of the corresponding disilenes >Si=Si<. The extent of these structural distortions in digermenes follows some general tendencies: electronegative substituents provoke notable elongation and weakening of the Ge=Ge bond, as well as *trans*-bending at the doubly bonded Ge centers, whereas electropositive substituents cause shortening and strengthening of the Ge=Ge bond and planarization at the doubly bonded Ge centers. Accordingly, the shortest Ge=Ge bond [*r*_Ge=Ge_ = 2.2576(5) Å] was found in the di(aryl)di(silyl)digermene **20**, whereas the longest one [*r*_Ge=Ge_ = 2.5087(7) Å] was detected in the di(aryl)di(bromo)digermene **6**, with the exceptionally bulky Bbt substituents (Table 1). Moreover, in line with what was mentioned above, tetra(silyl)digermene **9** has planar (the least *trans*-bent) geometry at its sp^2^-Ge centers (*θ* = 0.3°), whereas the greatest *trans*-bending was observed in the di(aryl)digermene **12** (*θ* = 45.0°). Twisting in digermenes is controlled by the substituent effect, to range from non-twisted Ge=Ge double bonds (*τ* = 0.0° in tetra(alkyl)digermene **1** and di(aryl)di(silyl)digermene **20**) to extraordinarily twisted (*τ* = 52.8° in tetra(silyl)digermene **14** with very bulky silyl substituents).

##### Cyclic Digermenes

To date, 22 neutral organogermanium compounds featuring an endocyclic Ge=Ge double bond, 14 three-membered rings, 4 four-membered rings, 3 five-membered rings, and 1 six-membered ring, are reported.

Three-Membered Ring Compounds

There are currently 14 heavy cyclopropene analogues incorporating a Ge=Ge double bond into the three-membered ring: 12 homonuclear cyclotrigermenes *cyclo*-[Ge_3_] and 2 heteronuclear 1*H*-siladigermirenes *cyclo*-[Si–Ge=Ge] (Table 2). Unlike their acyclic congeners, all of these cyclic digermenes are synthesized by special methods which are not outlined in Scheme 2.

**Scheme 5:**
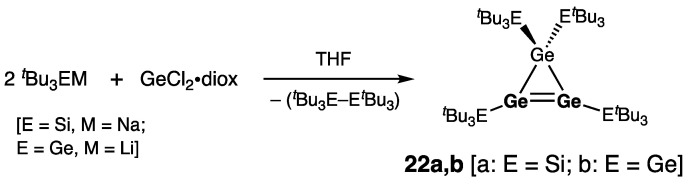
Synthesis of the first cyclotrigermenes **22a**,**b**.

The first isolable cyclotrigermenes **22a**,**b** were synthesized by Sekiguchi and coworkers by the reaction of *^t^*Bu_3_EM (a: E = Si, M = Na; b: E = Ge, M = Li) with GeCl_2_•diox (Scheme 5 and Table 2) [41]. Remarkably, one-electron oxidation of **22a** with [Ph_3_C]^+^[BPh_4_]^−^ produced tris(tri-*tert*-butylsilyl)cyclotrigermenylium tetraphenylborate [(*^t^*Bu_3_Si)_3_Ge_3_]^+^[BPh_4_]^−^, as the germanium analogue of aromatic 2*π*-electron cyclopropenylium ion [42].

**Scheme 6:**
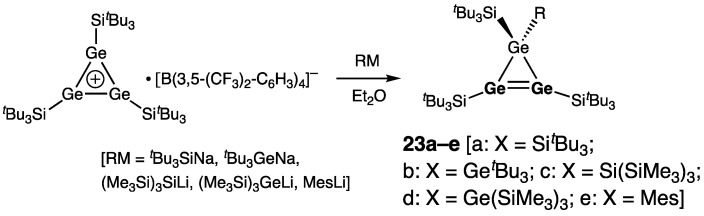
Synthesis of the unsymmetrically substituted cyclotrigermenes **23a**–**e**.

**Scheme 7:**
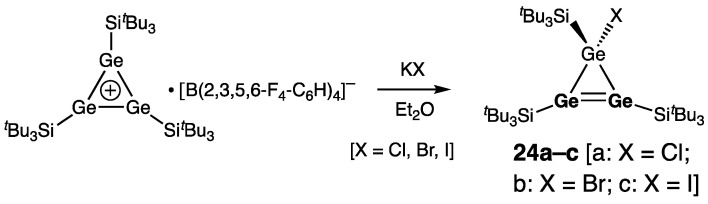
Synthesis of the halogen-substituted cyclotrigermenes **24a**–**c**.

Unsymmetrically substituted cyclotrigermenes **23a**–**e** (a: R = Si*^t^*Bu_3_; b: R = Ge*^t^*Bu_3_; c: R = Si(SiMe_3_)_3_; d: R = Ge(SiMe_3_)_3_; e: R = Mes) were later reported by the same authors prepared by the reaction of tris(tri-*tert*-butylsilyl)cyclotrigermenylium tetrakis[3,5-bis(trifluoromethyl)phenyl]borate with the alkali metal salts RM (R = silyl, germyl, aryl; M = alkali metal) (Scheme 6 and Table 2) [43]. Likewise, treatment of (tri-*tert*-butylsilyl)cyclotrigermenylium tetrakis(2,3,5,6-tetrafluorophenyl)borate with potassium halides KX (X = Cl, Br, I) formed halogen-substituted cyclotrigermenes **24a**–**c** (a: X = Cl, b: X = Br; c: X = I) (Scheme 7 and Table 2) [44].

**Scheme 8:**
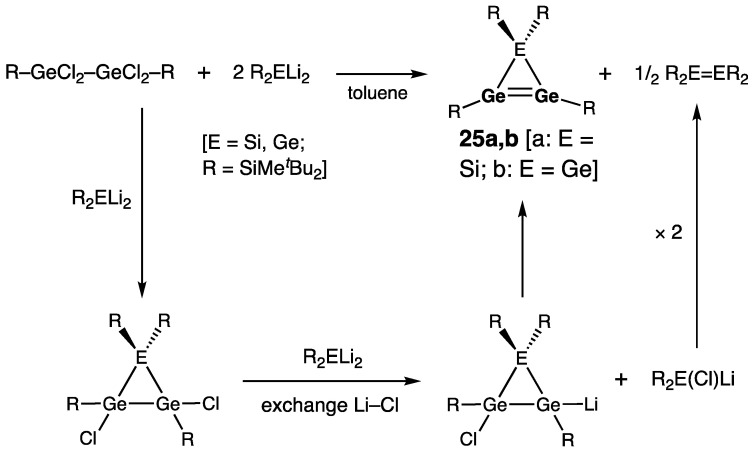
Synthesis of 1*H*-siladigermirene **25a** and 1*H*-trigermirene **25b**.

Lee, Sekiguchi, and coworkers reported novel three-membered ring cyclic digermenes, 1*H*-siladigermirene **25a** and 1*H*-trigermirene (cyclotrigermene) **25b**, synthesized by the reaction of 1,1,2,2-tetra(chloro)digermane R–GeCl_2_–GeCl_2_–R (R = SiMe*^t^*Bu_2_) with 1,1-di(lithio)silane R_2_SiLi_2_ or 1,1-di(lithio)germane R_2_GeLi_2_, respectively (Scheme 8 and Table 2) [45,46].

In due course, the solid-state thermolysis of the tetra(silyl)digermene (*^t^*Bu_2_MeSi)_2_Ge=Ge(SiMe*^t^*Bu_2_)_2_ **14**^27^ (170 °C, 1 h, evacuated sealed tube) was found to be an attractive alternative to the above-described preparation of 1*H*-trigermirene **25b**, improving the isolated yield of the latter up to 48% [47].

**Scheme 9:**
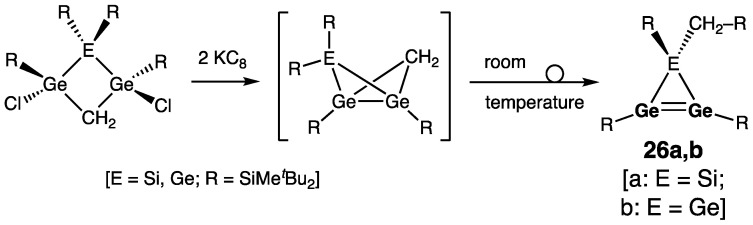
Synthesis of the alkyl-substituted heavy cyclopropene analogues **26a**,**b**.

The alkyl-substituted heavy cyclopropene analogues **26a**,**b** (a: E = Si; b: E = Ge), representing the nearest homologues of the above-described 1*H*-siladigermirene **25a** and 1*H*-trigermirene **25b**, being distinguished from them by only one CH_2_-unit, were prepared by Lee, Sekiguchi, and coworkers by the reductive dehalogenation of 1,3-di(chloro)cyclobutane derivatives (Scheme 9) [48]. The overall process was proposed to proceed via the transient bicyclo[1.1.0]butane derivatives, rapidly isomerizing at room temperature to the more stable heavy cyclopropenes **26a**,**b**.

In cyclotrigermenes, the Ge=Ge double bonds [*r*_Ge=Ge_ = 2.239(4)–2.2743(8) Å] are typically shorter than those of acyclic digermenes [*r*_Ge=Ge_ = 2.2576(5)–2.5087(7) Å] (Table 1 and Table 2). The longest Ge=Ge bonds were detected in halogen-substituted cyclotrigermenes **24a**–**c** [*r*_Ge=Ge_ = 2.2721(6)–2.2743(8) Å], and they were realized in terms of *π*_Ge=Ge_–σ*_Ge–X_ orbitals mixing facilitated by the electronegative halogen atoms X at the *sp*^3^-Ge [44]. Usually, cyclotrigermenes exhibited significant twisting about their Ge=Ge double bonds: *τ* = 8.1–51.0° (Table 2). Cyclotrigermene **25b** with very bulky *^t^*Bu_2_MeSi substituents showed a record twisting for the endocyclic Ge=Ge bond in heavy cyclopropenes (*τ* = 60.5°) [46].

Four-Membered Ring Compounds

Four neutral cyclobutene derivatives containing heavy group 14 elements and incorporating Ge=Ge double bond are currently known: one homonuclear (tetragermetene *cyclo*-[Ge_4_]) and three heteronuclear (disiladigermetene *cyclo*-[Si_2_Ge_2_], trigermetene *cyclo*-[Ge_3_C], and digermetene *cyclo*-[Ge_2_C_2_]).

**Scheme 10:**
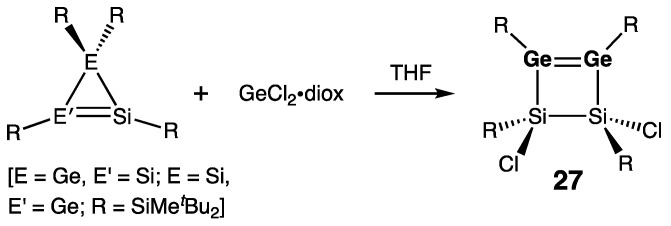
Synthesis of disiladigermetene **27**.

The very first compound of this type, namely disiladigermetene **27**, was reported by Lee, Sekiguchi, and coworkers, formed by the unexpected ring expansion of either 3*H*- or 1*H*-disilagermirenes [49] with GeCl_2_•diox (Scheme 10 and Table 2) [50]. The Ge=Ge double bond in **27** [*r*_Ge=Ge_ = 2.2911(4) Å] is one of the longest reported for the cyclic digermenes, whereas the endocyclic Si–Ge bonds are sizably shortened, and the exocyclic Si–Cl bonds are elongated. This was explained by the important extent of *π*_Ge=Ge_–σ*_Si–Cl_ negative hyperconjugation promoted by the presence of electronegative chlorine atoms and folding of the Si_2_Ge_2_-ring (folding angle = 28.3°). The geometry around the sp^2^-Ge atoms in **27** is only slightly pyramidal: ***Σ***_Ge_ = 357.2°/358.6°.

**Scheme 11:**
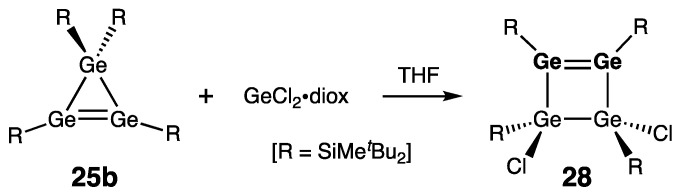
Synthesis of tetragermetene **28**.

Likewise, Lee, Sekiguchi, and coworkers found that the similar reaction of 1*H*-trigermirene **25b** with the GeCl_2_•diox yielded the first (and still the only known) homonuclear heavy cyclobutene analogue, tetragermetene **28** (Scheme 11 and Table 2) [51]. Structurally, tetragermetene **28** is similar to the above-described disiladigermetene **27**: *r*_Ge=Ge_ = 2.2993(5) Å (**28**) vs. 2.2911(4) (**27**), and ***Σ***_Ge_ = 354.4°/356.4° (**28**) vs. 357.2°/358.6° (**27**).

**Scheme 12:**
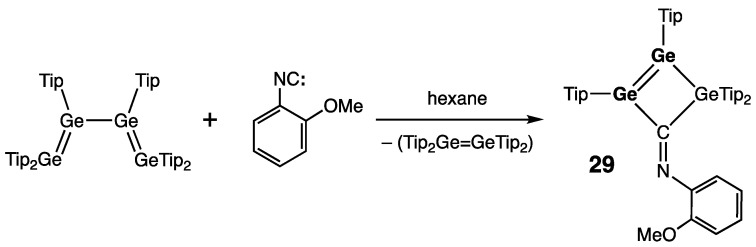
Synthesis of 1,2,3-trigermet-1-ene **29**.

Weidenbruch and coworkers developed an alternative approach toward heavy cyclobutene analogues by reacting tetra(germa)buta-1,3-diene Tip_2_Ge=Ge(Tip)–Ge(Tip)=GeTip_2_ with 2-methoxyphenyl isocyanide to produce 1,2,3-trigermet-1-ene **29**, featuring a germanium–germanium double bond within the Ge_3_C-skeleton (Scheme 12 and Table 2) [52]. The Ge=Ge bond in **29** was marginally shorter that those in disiladigermetene **27** and tetragermetene **28**: 2.2808(7) Å vs. 2.2911(4) and 2.2993(5) Å, respectively.

**Scheme 13:**
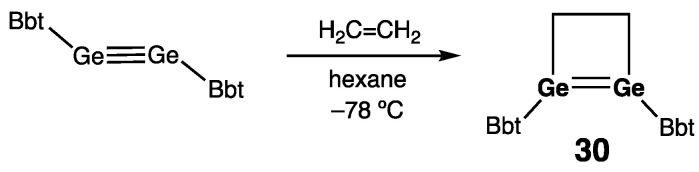
Synthesis of 1,2-digermet-1-ene **30**.

The four-membered ring 1,2-digermet-1-ene **30** with an endocyclic Ge=Ge double bond was prepared by Sasamori, Tokitoh, and coworkers by [2 + 2] cycloaddition of the stable digermyne BbtGe≡GeBbt (Bbt = 2,6-[(Me_3_Si)_2_CH]_2_-4-[(Me_3_Si)_3_C]-C_6_H_2_) and ethylene, (Scheme 13 and Table 2) [53]. In **30**, its remarkable ring strain and *trans*-bending of the substituents (*θ* = 39.5 and 39.7°) caused elongation and weakening of the Ge=Ge double bond [*r*_Ge=Ge_ = 2.4132(5) Å].

**Scheme 14:**
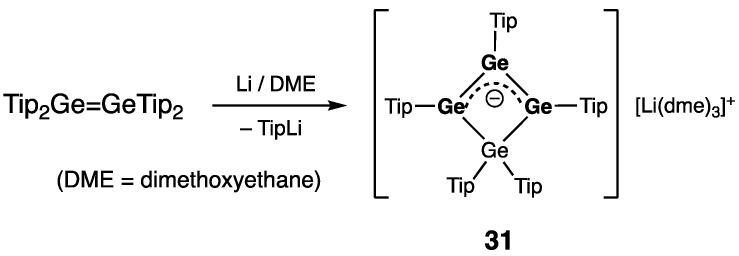
Synthesis of tetra(germa)cyclobutenyl anion **31**.

Except for the above-described neutral heavy cyclobutene analogues **27**–**30**, there are several four-membered ring compounds where the Ge=Ge double bond is a part of the tri(germa)allylic system (cationic, radical, or anionic). Thus, Weidenbruch and coworkers reported the tetra(germa)cyclobutenyl anion **31** unexpectedly produced by exhaustive reduction of Tip_2_Ge=GeTip_2_ with excess of lithium (Scheme 14) [54]. In **31**, the Ge_4_-ring is practically planar (sum of the internal bond angles = 360°), and within the tri(germa)allylic system, the Ge–Ge bonds length of 2.3679(6) Å is just in-between those of the typical Ge–Ge single and Ge=Ge double bonds.

**Scheme 15:**
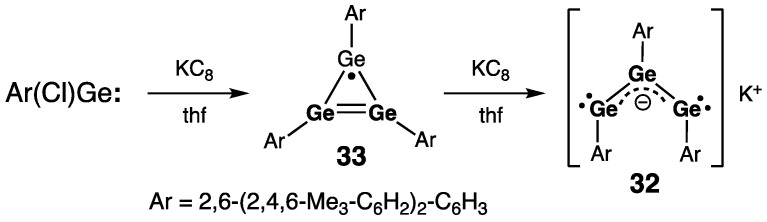
Synthesis of tri(germa)allyl anion **32**.

Cyclic compound **31** can be directly compared with acyclic tri(germa)allyl anion derivative **32**, reported by Power and coworkers and prepared by the reductive ring opening of a cyclotrigermenyl radical **33** with KC_8_ (Scheme 15) [55]. The germanium–germanium bond length [2.422(2) Å] in **32** is also intermediate between those of the Ge–Ge single and Ge=Ge double bonds, and the Ge_3_-array is characterized by a wide Ge–Ge–Ge bond angle [159.19(10)°].

**Scheme 16:**
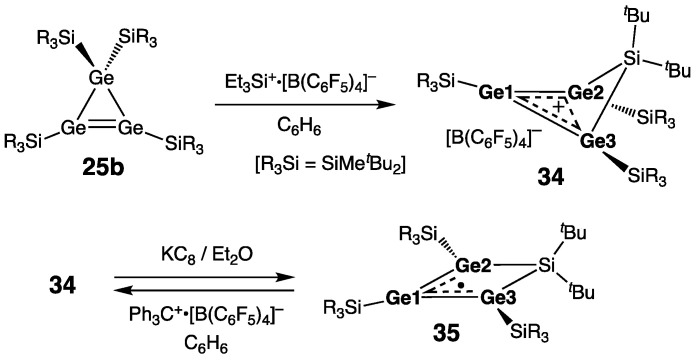
Synthesis of silatri(germa)cyclobutenylium ion derivative **34**.

Most recently, Lee, Sekiguchi, and coworkers synthesized silatri(germa)cyclobutenylium ion derivative **34** by the oxidative demethylation of the cyclotrigermene **25b** [45] with [Et_3_Si]^+^∙[B(C_6_F_5_)_4_]^−^ (Scheme 16) [56]. In **34**, the SiGe_3_ four-membered ring is strongly folded (folding angle 40.4°), thus enabling Ge2∙∙∙Ge3 *through-space* orbital interaction manifested in the short transannular Ge2–Ge3 distance of only 2.9346(3) Å. Overall, the structural peculiarities of **34** (ring folding and short transannular distance) testify to the important extent of its homoaromaticity. Accordingly, **34** is to be classified as a germanium analogue of the cyclobutenylium ion, i.e., a *homo*-tri(germa)cyclopropenylium ion. The homoaromaticity of **34** was further confirmed by the calculation of the nucleus-independent chemical shift (NICS) at 1 Å above the Ge_3_-ring center, which was diagnostically negative (−17.3) as a manifestation of the diatropic ring current. The “homoaromatization energy” of **34**, calculated as the barrier to inversion of the Ge_3_Si-ring (through the planar allylic-type cationic transition state lacking homoaromatic stabilization), was exceedingly low, i.e., only 3.7 kcal/mol [56]. In accordance with its homoaromaticity, **34** showed practically no alteration in the Ge–Ge bond lengths of its Ge_3_-fragment [*r*_Ge=Ge_ **=** 2.3327(3) Å (Ge1–Ge2) and 2.3400(3) Å (Ge1–Ge3)], and it showed essentially planar geometry at all skeletal Ge atoms [***Σ***_Ge_ = 360.0° (Ge1), 358.7° (Ge2), and 357.5° (Ge3)].

Upon the one-electron reduction of **34** with KC_8_, a free-radical species, namely silatri(germa)cyclobutenyl radical **35**, was cleanly formed (Scheme 16) [56]. The homoaromaticity of **34** is completely lost upon its reduction, which was seen in the remarkable flattening of the SiGe_3_-ring in **35** (folding angle was reduced from 40.4° in the starting **34** to only 6.9° in the resulting **35**) and great elongation of the Ge2∙∙∙Ge3 transannular distance [3.3315(4) Å in **35**, or 14% elongation compared with **34**]. Thus, **35** is to be classified as the allylic free radical, featuring the unpaired electron delocalized over the two terminal Ge atoms, [∙Ge2–Ge1=Ge3 ↔ Ge2=Ge1–Ge3∙]. Accordingly, the lengths of both Ge–Ge bonds in **35** are intermediate between those for the single and double bonds [2.3458(3) Å (Ge1–Ge2) and 2.3206(3) Å (Ge1–Ge3)], and all Ge atoms maintain essential planarity of their geometry upon reduction [***Σ***_Ge_ = 358.6° (Ge1), 355.2° (Ge2), and 359.9° (Ge3)]. The EPR resonance *g*-value of **35** (2.0227) is in the range typical for the silyl-substituted Ge-centered free radicals. In the Ge_3_-unit of **35**, the hyperfine coupling constants (hfcc) for the terminal Ge nuclei are markedly greater than that for the central Ge nucleus: *a*(^73^Ge2) [or *a*(^73^Ge3)] = 1.54 mT [or 1.44 mT] vs. *a*(^73^Ge1) = 0.59 mT. This observation agrees well with the allylic radical formulation of **35**, in which the odd electron is mostly localized at the Ge2 and Ge3 termini. Given that the small values of the ^73^Ge hfcc in **35** imply the location of its unpaired electron in the orbital of *π*-symmetry, **35** should be categorized as a *π*-radical. The allylic free radical **35** can be compared with the cyclotrigermenyl radical **33** prepared by Power and coworkers by the stoichiometric reduction of the aryl(chloro)germylene Ar(Cl)Ge: [Ar = 2,6-(2,4,6-Me_3_-C_6_H_2_)_2_-C_6_H_3_] with KC_8_ (Scheme 15) [55]. In the cyclotrigermenyl radical **33**, the average Ge–Ge bond distance within the Ge_3_-ring of 2.35(7) Å is comparable with those of the silatri(germa)cyclobutenyl radical **35** [2.3458(3) Å and 2.3206(3) Å]. The EPR characteristics of **33** are also comparable to those of **35**: *g* = 2.0069 [2.0227 in **35**], and *a*(^73^Ge) = 1.6 mT [*a*(^73^Ge2) or *a*(^73^Ge3) = 1.54 or 1.44 mT in **35**].

Five-Membered Ring Compounds

**Scheme 17:**
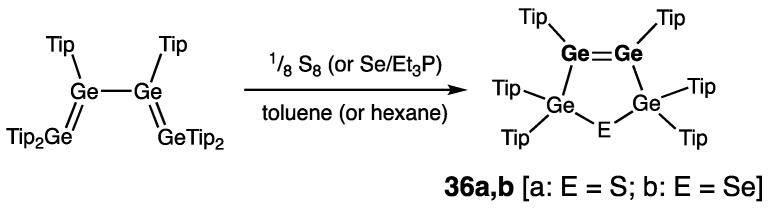
Synthesis of heavy cyclopentene analogues **36a**,**b** with endocyclic Ge=Ge double bonds.

Three compounds of this type with the Ge=Ge double bond within the five-membered ring skeleton have been reported to date. Weidenbruch and coworkers synthesized the first two representatives by [1 + 4] cycloaddition of sulfur/or selenium to the tetra(germa)buta-1,3-diene Tip_2_Ge=Ge(Tip)–Ge(Tip)=GeTip_2_, forming thia- or selena-tetra(germa)cyclopentenes **36a**,**b** (Scheme 17 and Table 2) [52,57]. The structural features of heavy cyclopentenes **36a**,**b** are typical for the cyclic digermenes: *r*_Ge=Ge_ = 2.2841(5) Å (**36a**) and 2.2975(5) Å (**36b**), and ***Σ***_Ge_ = 348.4°/351.4° (**36a**) and 349.2°/349.9° (**36b**); and Ge_4_E rings are practically planar (sums of the internal bond angles are 539.5° (**36a**) and 537.7° (**36b**)).

**Scheme 18:**
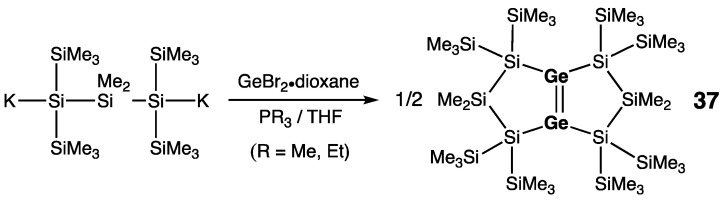
Synthesis of bicyclic digermene **37**.

The latest example of the five-membered ring cyclic digermene, namely bicyclic digermene **37** with a bridging Ge=Ge double bond, was prepared by Marschner and coworkers; it was unexpectedly formed by the reaction of 1,3-di(potassio)trisilane with GeBr_2_∙dioxane and PEt_3_ (Scheme 18) [58]. In **37**, the Ge=Ge double bond revealed the structural parameters that are characteristic for the cyclic digermenes: shortened bond [*r*_Ge=Ge_ = 2.2663(9) Å], negligible *trans*-bending of substituents [*θ* = 2.5°], and moderate twisting [*τ* = 16.2°]. Upon its electrochemical (CV) reduction, digermene **37** showed two reversible reduction waves corresponding to generation of anion-radical and dianion.

Six-Membered Ring Compounds

**Scheme 19:**
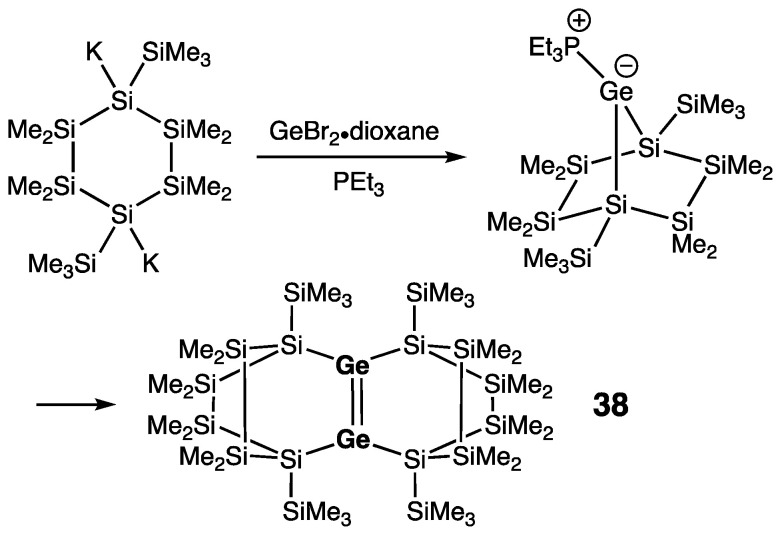
Synthesis of tetracyclic digermene **38**.

Only one “heavy cyclohexene” derivative has been reported to date. Marschner and coworkers prepared this tetracyclic digermene **38** with an endocyclic Ge=Ge double bond bridging polycyclic scaffold (Scheme 19) [58]. Digermene **38** was available via the synthetic strategy applied for the preparation of the above-described bicyclic digermene **37**, namely by the reaction of 1,4-di(potassio)cyclohexasilane with GeBr_2_∙dioxane in the presence of PEt_3_. The metric parameters of **38** are comparable to those of **37**: *r*_Ge=Ge_ = 2.2896(6) Å, *θ* = 2.1/8.3°, and *τ* = 5.2°.

### 2.2. Heteronuclear Derivatives

#### 2.2.1. Germenes >Ge=C<

**Scheme 20:**
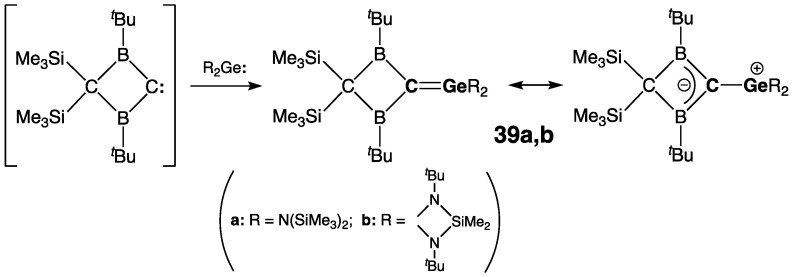
Synthesis of stable germenes **39a**,**b**.

The first isolable germenes were independently synthesized in 1987 by the groups of Berndt and Escudié. Berndt and coworkers prepared germenes **39a**,**b** by the coupling of electrophilic “cryptocarbene” and bis(amino)germylenes (Scheme 20) [59]. The formulation of **39a**,**b** as germenes Ge=C was supported by the observation of low-field resonance for their doubly bonded C atoms [*δ*(^13^C) = 115.0 ppm (**39a**) and 93.2 ppm (**39b**)] and rather short Ge=C bond [*r*_Ge=C_ = 1.827(4) Å (**39a**)]. The Ge=C double bond in **39a** was significantly twisted [*τ* = 36°], although the sp^2^-Ge center featured a planar geometry [***Σ***_Ge_ = 359.9°]. The spectroscopic and crystallographic data of **39** are indicative of the substantial contribution of the ylide resonance structure with a positive charge on the Ge atom and a negative charge delocalized over the B–C–B allylic fragment of the B_2_C_2_ four-membered ring (Scheme 20) [59]. This was exemplified by the shielding of the B atoms [*δ*(^11^B) = 66.0 ppm (**39a**) and 65.0 ppm (**39b**)] and shortening of the cyclic sp^2^-C–B bonds in **39a** [*r*_C–B_ = 1.534(7)/1.523(6) Å].

**Scheme 21:**
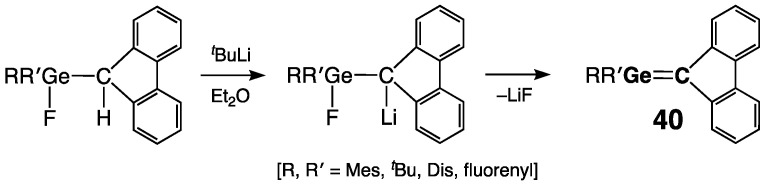
Synthesis of stable germene **40**.

Escudié and coworkers applied another approach toward their stable dimesityl(fluorenylidene)germene **40**: dehydrofluorination of the (fluorenyl)fluorogermane percursor by its lithiation with *^t^*BuLi with subsequent elimination of LiF from the intermediate lithium salt (Scheme 21, R = R′ = Mes) [60]. In **40**, the Ge=C double bond of 1.803(4) Å (mean value for the two crystallographically independent molecules) was notably shorter than the typical Ge–C single bonds and even shorter than that in **39a**, being practically undistorted [***Σ***_Ge_ = ***Σ***_C_ = 360.0°, *τ* = 6°] [61]. Except for the steric protection of the Ge=C bond by the bulky Mes groups, extra stabilization in **40** results from the contribution of the charge-separated Ge*^δ^*^+^=C*^δ^*^−^ resonance form enabled by the effective aromatic delocalization of the negative charge over the cyclopentadiene fragment of the fluorenylidene group.

Applying the same synthetic protocol, Escudié and coworkers prepared other stable germenes **40**, as outlined in Scheme 21: R = R′ =Dis [62]; R = Dis, R′ = Mes [62]; R = R′ = fluorenyl [63]; and R = *t*-Bu, R′ = fluorenyl [63]. They also reported generation of the halogen-substituted germenes (Me_5_C_5_)(X)Ge=CR_2_ [CR_2_ = fluorenylidene] **40** (Scheme 21: R = C_5_Me_5_; R′ = F, Cl) by *β*-elimination of Me_3_SiF from the (Me_5_C_5_)(X)FGe–(Me_3_Si)CR_2_ precursors [64]. Lacking resonance stabilization, dimesityl(neopentyl)germene Mes_2_Ge=CH–CH_2_*^t^*Bu **41** was synthesized by Couret and coworkers via the addition of *^t^*BuLi to Mes_2_(F)Ge–CH=CH_2_, followed by *β*-elimination of LiF from the intermediate lithium salt Mes_2_(F)Ge–CH(Li)–CH_2_*^t^*Bu [65].

**Scheme 22:**
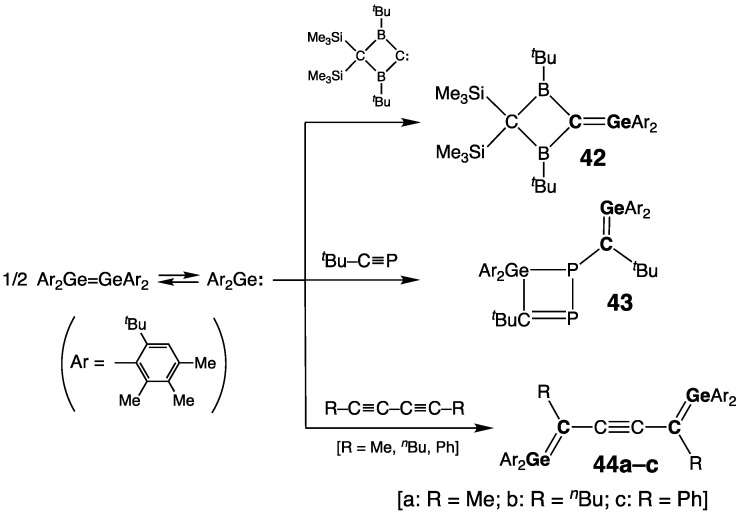
Synthesis of germenes **42**–**44**.

A series of stable germenes **42**–**44**, prepared from a germylene Ar_2_Ge: (Ar = 2-*^t^*Bu-4,5,6-Me_3_-C_6_H), were reported by Weidenbruch and coworkers (Scheme 22) [17,66,67,68,69]. The germene **42**, similar to Berndt’s germene **39a**, disclosed a short [*r*_Ge=C_ = 1.845(10) Å] and remarkably twisted [*τ* = 33°] Ge=C bond, and planar geometry at both sp^2^-Ge and sp^2^-C atoms [***Σ***_Ge_ = ***Σ***_C_ = 359.9°] [66]. The reaction of two equivalents of germylene Ar_2_Ge: with two equivalents of phosphaalkyne *^t^*Bu–C≡P unexpectedly produced a germene **43** with an exocyclic Ge=C double bond [*r*_Ge=C_ = 1.833(4) Å] (Scheme 22) [67]. A series of bis(germenes) connected via acetylene spacer, **44a**–**c**, were synthesized by the double [1 + 2] cycloaddition of germylenes Ar_2_Ge: to the C≡C bonds of bis(alkynes), followed by the ring-opening isomerization of the transient bis(germirenes) (Scheme 22) [17,68,69]. The length of the Ge=C double bond in **44a**–**c** was in the range expected for germenes: 1.819(6) Å in **44a** [17], 1.819(2) Å in **44b** [68], and 1. 840(4) Å in **44c** [69]. The observable conjugation between the terminal Ge=C bonds and the central C≡C bond in **44a**–**c** was seen in the diagnostic bathochromic shift of their longest wavelength UV absorptions [500 nm (**44a**) [17], 518 nm (**44b**) [68], and 595 nm (**44c**) [69], as well as in the shortening of the central C–C bonds in the Ge=C–C≡C fragments.

**Scheme 23:**
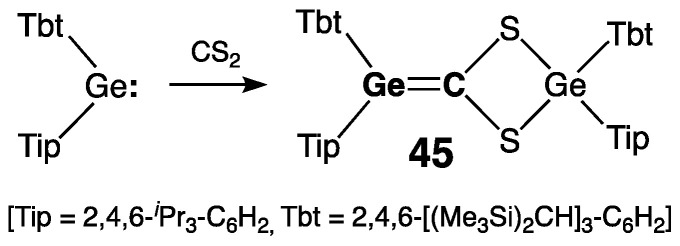
Synthesis of the germene **45** with the shortest Ge=C double bond.

Two stable germenes, exemplifying the shortest and the longest extremes for the range of Ge=C bond lengths, are of particular interest. The germene with the shortest Ge=C double bond, **45**, was prepared by Okazaki and coworkers by the reaction of di(aryl)germylene Tbt(Tip)Ge: (generated by the reduction of di(bromo)germane Tbt(Tip)GeBr_2_ with lithium naphthalenide) with CS_2_ (Scheme 23) [70]. The length of the Ge=C double bond in **45** was only 1.771(16) Å, a value which was substantially smaller than those of Berndt’s germene **39a** (1.827(4) Å) [59] and Escudié’s germene **40** (1.803(4) Å) [61]. Furthermore, the Ge=C bond in **45** showed no signs of structural distortion, exhibiting a trigonal-planar geometry at both sp^2^-Ge and sp^2^-C centers [***Σ***_Ge_ = 359.7°, ***Σ***_C_ = 360.0°] and a negligible twisting [*τ* = 4°].

**Scheme 24:**
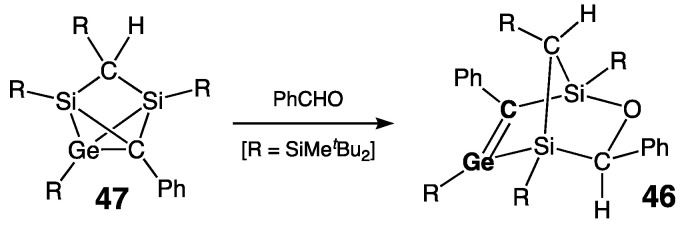
Synthesis of the germene **46** with the longest Ge=C double bond.

The germene **46** with the longest ever reported Ge=C bond [*r*_Ge=C_ = 1.895(3) Å] was reported by Lee, Sekiguchi, and coworkers in 2002 [71]. This endocyclic Ge=C bond was embedded into the norbornene bicyclic skeleton of **46**, which was unexpectedly formed by the reaction of 2,4-disila-1-germatricyclo[2.1.0.0^2,5^]pentane cage **47** with benzaldehyde (Scheme 24). The major factor responsible for such extraordinary stretching of the germanium–carbon bond is most likely a steric repulsion between the bulky substituents. Nevertheless, despite this exceptional steric crowding around the double bond, the Ge=C bond in **46** revealed practically no structural deformations, neither pyramidalization at the doubly bonded Ge and C atoms nor twisting of the Ge=C bond: ***Σ***_Ge_ = 359.8°/***Σ***_C_ = 358.9°, *τ* = 7.1°.

**Scheme 25:**
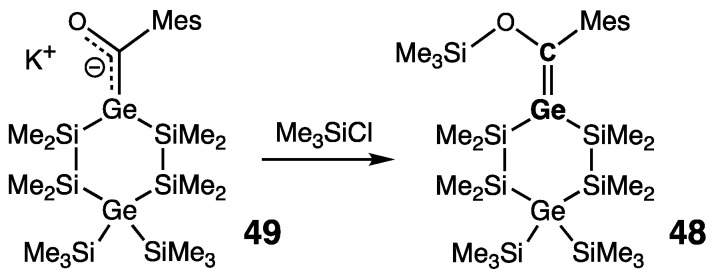
Synthesis of the Brook-type germene **48**.

The latest representative of isolable germenes, namely the first stable Brook-type germene **48** with an exocyclic Ge=C double bond, was prepared by Haas and coworkers [72]. This *O*-silylated germene **48** was prepared by the reaction of Me_3_SiCl with the stable germenolate **49**, predominantly exhibiting acyl germyl anion character with a negatively charged Ge, Ge–C single bond, and C=O double bond (Scheme 25). Spectroscopic and structural characteristics of **48** are typical for the isolable germenes: strongly deshielded sp^2^-C atom [*δ*(^13^C) = 210.0 ppm]; short [*r*_Ge=C_ = 1.835(2) Å], pyramidalized at Ge [***Σ***_Ge_ = 351.7° (*cf.*: ***Σ***_C_ = 360.0°)], and twisted (torsional angles: O–C=Ge–Si = 10.0° and C_Mes_–C=Ge–Si = 18.1°) Ge=C double bond.

#### 2.2.2. Silagermenes >Si=Ge<

The first ever reported silagermene, metastable Mes_2_Si=GeMes_2_, was thermally or photochemically generated from hexa(mesityl)siladigermirane precursor, *cyclo*-(Mes_2_Si–GeMes_2_–GeMes_2_), by Baines and coworker [73]. Stable in solution only below −70 °C, Mes_2_Si=GeMes_2_ was identified by low-temperature NMR and UV measurements (*δ*(^29^Si) = 80.6 ppm and 414 nm (*π*_Si=Ge_–π*_Si=Ge_), respectively), as well as by the quenching with methanol forming 1,2-addition product Mes_2_Ge(H)–Si(OMe)Mes_2_. Above −70 °C, Mes_2_Si=GeMes_2_ underwent irreversible 1,2-migration of Mes-group from Ge to Si generating transient mesityl(silyl)germylene Mes(Mes_3_Si)Ge:.

**Scheme 26:**
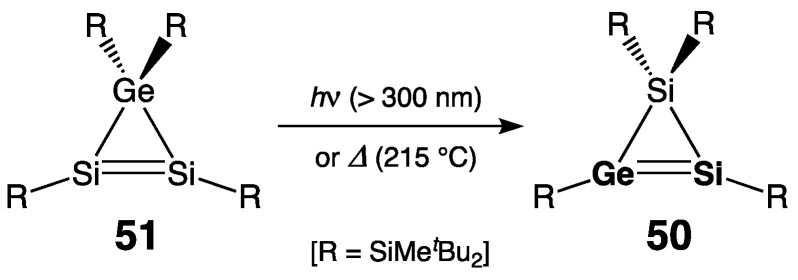
Synthesis of the first isolable silagermene **50**.

The first isolable silagermene, 1*H*-disilagermirene **50**, with a cyclic Si=Ge double bond within the three-membered ring GeSi_2_-skeleton, was reported by Lee, Sekiguchi, and coworkers in 2000 [49]. Moreover, **50** was quantitatively available from 3*H*-disilagermirene precursor **51** by either thermal or photochemical isomerization, driven by the thermodynamic preference for the silagermene >Si=Ge< over the disilene >Si=Si< (Scheme 26). In **50**, the sp^2^-Si center was expectedly deshielded [*δ*(^29^Si) = 100.7 ppm], and the Si=Ge double bond was notably twisted (torsional angle R–Si=Ge–R = 40.3°).

**Scheme 27:**
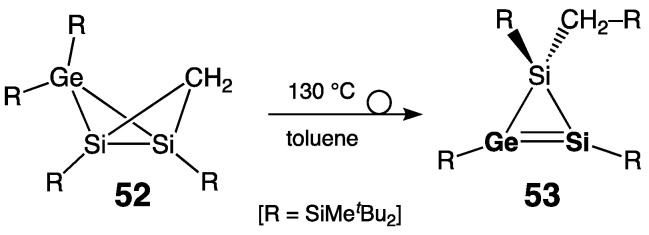
Synthesis of alkyl-substituted 1*H*-disilagermirene **53**.

Lee, Sekiguchi, and coworkers later developed another approach toward isolable silagermenes by the thermal isomerization of disilagermabicyclo[1.1.0]butane **52,** forming novel 1*H*-disilagermirene **53**, as the nearest homologue of **50**, distinguished from the latter by only a CH_2_ unit (Scheme 27) [48]. Compared to **50**, **53** revealed a more deshielded sp^2^-Si center: *δ*(^29^Si) = 100.7 ppm and 126.6 ppm, respectively. Bearing RCH_2_-substituent at the sp^3^-Si atom, 1*H*-disilagermirene **53** is the first representative of the alkyl-substituted cyclopropene analogues of the heavy group 14 elements.

The other three silagermenes, namely (*^t^*Bu_2_MeSi)_2_Si=GeMes_2_ **54** [74], Mes_2_Si=Ge(SiMe*^t^*Bu_2_)_2_ **55** [75], and (*^t^*Bu_3_Si)_2_Si=GeMes_2_ **56** [76], were available by the reaction of 1,1-di(lithio)derivatives (*^t^*Bu_2_MeSi)_2_SiLi_2_, (*^t^*Bu_2_MeSi)_2_GeLi_2_, and (*^t^*Bu_3_Si)_2_SiLi_2_ with di(aryl)dichlorides Mes_2_GeCl_2_, Mes_2_SiCl_2_, and Mes_2_GeCl_2_, respectively. The silagermene **55** showed a characteristic low-field signal of its sp^2^-Si atom [*δ*(^29^Si) = 146.9 ppm], whereas silagermenes **54** and **56** exhibited unusually shielded sp^2^-Si centers [*δ*(^29^Si) = 22.4 ppm and 18.7 ppm, respectively]. This striking spectroscopic distinction resulted from the differing substitution pattern in **54** and **56** (donating groups on Si and withdrawing groups on Ge), which alters the natural Si*^δ^*^+^=Ge*^δ^*^−^ bond polarity (like in **55**) into a reversed one Si*^δ^*^−^=Ge*^δ^*^+^ (like in **54** and **56**). Only silagermene **56** was crystallographically identified as featuring peculiar properties caused by the exceptional steric crowding of its *^t^*Bu_3_Si-substituents: rather long [*r*_Si=Ge_ = 2.2769(8) Å] and markedly twisted [*τ* = 24.7°] Si=Ge double bond with planar geometry at the sp^2^-Si and sp^2^-Ge atoms [***Σ***_Si_ = ***Σ***_Ge_ = 360°]. Upon thermolysis at 100 °C, **56** quantitatively isomerized to a symmetrically substituted silagermene (*E*)-[*^t^*Bu_3_Si(Mes)Si=Ge(Mes)Si*^t^*Bu_3_] [76].

Iwamoto, Kira, and coworkers reported identically substituted silagermene, (*^t^*BuMe_2_Si)_2_Si=Ge(SiMe_2_*^t^*Bu)_2_ **57**, prepared by the reductive debromination of 1,2-di(bromo)silagermane Br(*^t^*BuMe_2_Si)_2_Si–Ge(SiMe_2_*^t^*Bu)_2_Br with sodium in toluene [22]. As with other stable silagermenes, **57** manifested a low-field resonance for its sp^2^-Si center [*δ*(^29^Si) = 144.0 ppm], a short (compared to other silagermenes) [*r*_Si=Ge_ = 2.2208(4) Å] and almost undistorted [*τ* = 7.5°] Si=Ge double bond, and practically planar geometry at the doubly bonded Si and Ge atoms [*θ* = 0.6°].

The ”inorganic ethylene” donor–acceptor *push–pull* complex, {[IPr:]→SiH_2_–H_2_Ge:→[W(CO)_5_]} **58**, in which the “SiH_2_–H_2_Ge” fragment is complexed with both Lewis base [IPr (IPr = 1,3-bis(2,6-diisopropylphenyl)-2*H*-imidazol-2-ylidene)] and Lewis acid [W(CO)_5_], was reported by Rivard and coworkers [77]. Moreover, **58** was synthesized by the reaction of the di(chloro)silylene complex [IPr∙SiCl_2_] [78] and [Cl_2_Ge∙W(CO)_5_], giving at first [IPr∙SiCl_2_–Cl_2_Ge∙W(CO)_5_] a complex that was subsequently reduced with LiAlH_4_ to form the final complex, {[IPr:]→SiH_2_–H_2_Ge:→[W(CO)_5_]} **58**. Given the strongly shielded Si nucleus [*δ*(^29^Si) = –71.9 ppm (triplet, ^1^*J*_Si–H_ = 192.2 Hz)] and very long Si–Ge bond of 2.3717(14) Å, the central Si–Ge interaction in **58** is best classified as a single, rather than a double, bond. This was supported by the computations on the model compound (Me in place of the real 2,6-*^i^*Pr_2_-C_6_H_3_ substituents on N atoms in IPr ligand; B3LYP/cc-pVDZ-pp), which yielded WBI_Si–Ge_ value of only 0.88, thus suggesting Si–Ge single-type bonding.

**Scheme 28:**
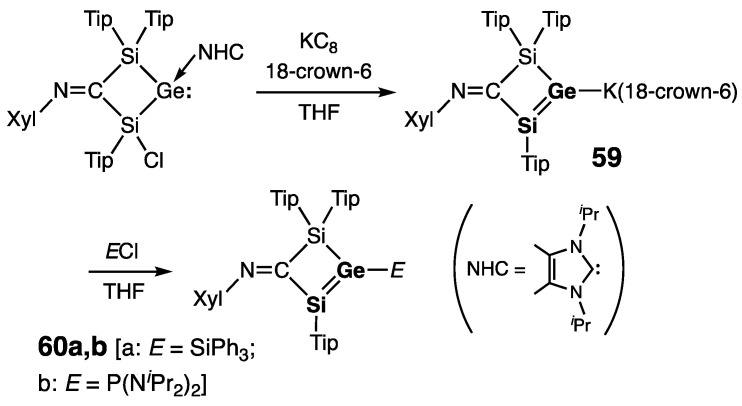
Synthesis of anionic silagermenide **59** and neutral silagermenes **60a**,**b**.

The latest example of the stable silagermenes was very recently reported by Scheschkewitz and coworkers. Their cyclic potassium silagermenide **59**, representing a Si=Ge analogue of a vinyl anion, was synthesized by the reduction of a germylene–NHC complex with KC_8_ (Scheme 28) [79]. The spectroscopic and structural features of **59** conform well to those of other stable silagermenes: characteristically deshielded sp^2^-Si center [*δ*(^29^Si) = 142.9 ppm (in C_6_D_6_) and 138.5 ppm (in thf-*d*_8_)] and short Si=Ge bond [*r*_Si=Ge_ = 2.2590(3) Å]. Moreover, the GeSi_2_C-ring in **59** is nearly planar, with a negligible folding of 1.9°. According to the crystallographic and UV-spectroscopic data, the *π*-conjugative interaction between the endocyclic Si=Ge double bond and exocyclic C=N double bond in **59** is notable with the calculated value for the *π*_Si=Ge_–π*_C=N_ interaction energy of 23.6 kcal mol^−1^. Silagermenide **59** can be functionalized at the anionic Ge with the appropriate electrophiles to form novel neutral Ge-substituted silagermenes **60a**,**b** (Scheme 28) [79]. Similar to the starting silagermene **59**, both (silyl)silagermene **60a** [*E* = SiPh_3_] and (phosphanyl)silagermene **60b** [*E* = P(N*^i^*Pr_2_)_2_] showed low-field resonances for their sp^2^-Si atoms [*δ*(^29^Si) = 136.6 ppm (singlet) and 104.5 ppm (doublet, ^2^*J*_Si–P_ = 9.8 Hz), respectively], and short Si=Ge bonds [*r*_Si=Ge_ = 2.2020(2) and 2.2252(4) Å, respectively]. The GeSi_2_C-ring in **60a** is practically planar (folding angle = 0.2°), and the geometry around the Ge atom is slightly pyramidal (***Σ***_Ge_ = 357.3°).

Silagermenes, in which the Si=Ge double bond is a part of the 1,3-diene (>Si=Ge–C=C<), allene (>Si=Ge=Si</>Ge=Si=Ge<), or vinylidene (>Si=Ge:) system, are discussed separately in Section 4.2.1, Section 5.1, and Section 6.2.1, respectively.

#### 2.2.3. Germastannenes >Ge=Sn<

The first reported transient germastannene, [Mes_2_Ge=SnTip_2_], was generated via the dehydrofluorination of Mes_2_(H)Ge–Sn(F)Tip_2_ by *^t^*BuLi by Escudié and coworkers [80]. Decomposing at room temperature, this germastannene was proved to be as such on the basis of its low-field ^119^Sn NMR resonance [360.0 ppm, −20 °C] and trapping reactions with MeOH and PhCHO.

As for the stable germastannenes, four of them were reported in 2003/2004. The first three were synthesized by the research groups of Weidenbruch and Sekiguchi in 2003. Thus, Weidenbruch and coworkers prepared the first structurally authenticated germastannene, Tip_2_Ge=SnTip_2_ **61**, by a one-pot low temperature reaction of TipMgBr, GeCl_2_•diox and SnCl_2_ [81]. Expectedly, germastannene **61** revealed low-field resonance of its sp^2^-Sn center [*δ*(^119^Sn) = 268.0 ppm], short Ge=Sn bond [*r*_Ge=Sn_ = 2.5065(5) Å] that was substantially shorter than the typical Ge–Sn single bonds, and remarkable *trans*-bending of the substituents [*θ* = 30.2° (at Ge) and 43.3° (at Sn)]. Being stable in the solid form, **61** decomposed in solution via dissociation of the Ge=Sn double bond to give a cyclotristannane *cyclo*-(Tip_2_Sn)_3_ and a digermene Tip_2_Ge=GeTip_2_.

**Scheme 29:**
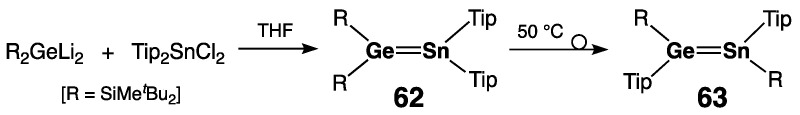
Synthesis of the unsymmetrically substituted germastannene **62** and its thermal isomerization to the symmetrically substituted germastannene **63**.

Another stable germastannene, unsymmetrically substituted (*^t^*Bu_2_MeSi)_2_Ge=SnTip_2_ **62**, was synthesized in 2003 by Sekiguchi and coworkers by the coupling of 1,1-di(lithio)germane (*^t^*Bu_2_MeSi)_2_GeLi_2_ and di(aryl)di(chloro)stannane Tip_2_SnCl_2_ (Scheme 29) [82]. In the absence of X-ray data, **62** was identified by its characteristic low-field tin resonance [*δ*(^119^Sn) = 525.1 ppm]. Upon thermolysis at 50 °C, **62** quantitatively isomerized to a symmetrically substituted germastannene, (*E*)-[Tip(*^t^*Bu_2_MeSi)Ge=Sn(SiMe*^t^*Bu_2_)Tip] **63** (Scheme 29) [82]. A concerted isomerization pathway involving simultaneous 1,2-migration of the silyl and aryl groups in the starting germastannene **62** was proposed, based on the negative value of the activation entropy (Δ*S*^‡^ = –12.0 cal/K·mol). The doubly bonded Sn atom in isomeric **63** was more shielded than that in the starting **62**, 373.4 ppm vs. 525.1 ppm, as a result of their different substitution patterns. The preliminary crystallographic data of **63** revealed the *trans*-bending of substituents at the Ge=Sn bond with *θ* angles of ca. 28.0°.

**Scheme 30:**
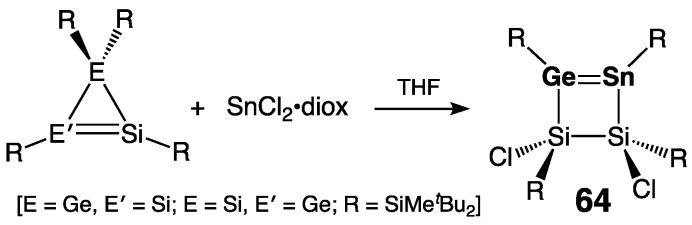
Synthesis of cyclic germastannene **64**.

The only currently known cyclic germastannene, ^3^*Δ*-1,2,3,4-disilagermastannetene **64**, featuring an endocyclic Ge=Sn double bond incorporated into the four-membered ring skeleton, was synthesized by Lee, Sekiguchi and coworkers in 2004 [83]. Moreover, **64** was readily available by the ring expansion of either 3*H*- or 1*H*-disilagermirenes [49] with SnCl_2_•diox (Scheme 30). As is typical for germastannenes, the doubly bonded Sn atom was diagnostically deshielded [*δ*(^119^Sn) = 439.3 ppm]. In a sharp contrast to the acyclic tetra(aryl)germastannenes Mes_2_Ge=SnTip_2_ [80] and Tip_2_Ge=SnTip_2_ 61 [81], cyclic tetra(silyl)germastannene **64** was indefinitely stable both in the solid state and in solution, showing no signs of dissociation of its >Ge=Sn< double bond into the germylene >Ge: and stannylene >Sn:. The unexpected high thermal stability of **64** was assigned to the influence of its *σ*-donating silyl substituents, further enhanced by the proposed *π*_Ge=Sn_→σ*_Si–Cl_ orbital mixing lowering the *π*_Ge=Sn_-orbital energy level and thus stabilizing the HOMO of the molecule [83].

Since 2004, no stable germastannenes >Ge=Sn< were reported.

## 3. Heavy Analogues of Alkynes

### 3.1. Homonuclear Derivatives

#### Digermynes –Ge≡Ge–

The first digermyne Ar′–Ge≡Ge–Ar′ [Ar′ = 2,6-(2,6-*^i^*Pr_2_-C_6_H_3_)_2_-C_6_H_3_] **65** was synthesized and structurally characterized in 2002 by Power and coworkers via the reduction of the (chloro)germylene Ar′(Cl)Ge: with potassium [84]. The C_Ar′_–Ge–Ge–C_Ar′_ core in digermyne **65** was planar and *trans*-bent with the Ge–Ge–C_Ar′_ bond angle of 128.7°. The Ge–Ge bond was rather short [*r*_Ge≡Ge_ = 2.2850(6) Å], which is indicative of its considerable multiply bonded character. On the other hand, accumulation of the lone pair electron density at the Ge atoms results in the *trans*-bending of their substituents, and consequently in the decrease (compared to the ideal triple bonding) of the Ge–Ge bond order and bond strength.

Following this report, Power’s group prepared a series of stable terphenyl-substituted digermynes, Ar*–Ge≡Ge–Ar* [Ar* = 2,6-(2,4,6-*^i^*Pr_3_-C_6_H_2_)_2_-C_6_H_3_] **66** [85,86], Ar″–Ge≡Ge–Ar″ [Ar″ = 4-Cl-{2,6-(2,6-*^i^*Pr_2_-C_6_H_3_)_2_}-C_6_H_2_] **67** [87], Ar‴–Ge≡Ge–Ar‴ [Ar‴ = 4-Me_3_Si-{2,6-(2,6-*^i^*Pr_2_-C_6_H_3_)_2_}-C_6_H_2_] **68** [87], and Ar**–Ge≡Ge–Ar** [Ar** = 3,5-*^i^*Pr_2_-{2,6-(2,4,6-*^i^*Pr_3_-C_6_H_2_)_2_}-C_6_H] **69** [87], which were uniformly prepared by the reduction of the corresponding aryl(chloro)germylenes with K or KC_8_. The digermyne **66** was characterized by NMR spectroscopy, elemental analysis, and its reaction with 2,3-dimethyl-1,3-butadiene [85,86]. All structurally characterized digermynes **67**–**69** displayed features similar to those of the first digermyne **65**, namely planar [C_Ar_–Ge–Ge–C_Ar_ torsional angles = 180° (for **67** and **68**) and 165.8° (for **69**)] and *trans*-bent [*trans*-bent Ge–Ge–C_Ar_ angles = 124.2° (for **67**), 128.4° (for **68**), and 136.1° (for very crowded **69**)] C_Ar_–Ge–Ge–C_Ar_ core arrays [84,87]. The Ge–Ge bonds in digermynes **67**–**69** were short [*r*_Ge≡Ge_ = 2.3071(3) Å (for **67**), 2.2438(8) Å (for **68**), 2.2125(13) Å (for **69**)], being comparable to those in digermenes >Ge=Ge<. The different Ge–Ge bond lengths in digermynes **65** and **67**–**69** can be understood in terms of the substituents’ electronic effects. Thus, digermynes **68** and **69** with the shortest Ge–Ge bonds feature electron donating alkyl or silyl substituents on the central aryl rings, whereas digermyne **65** (which has no such electron donating groups) and digermyne **67** (with electron withdrawing Cl substituents) have notably longer Ge–Ge bonds. Given that the electron donating groups typically reduce the extent of the *trans*-bending, whereas electron withdrawing groups having the opposite effect, it is reasonable that a smaller bending leads to a higher bond order and a shorter Ge–Ge bond, and vice versa.

Jones and coworkers reported bis(amido)digermyne R–Ge≡Ge–R {R = N(Si*^i^*Pr_3_)[2,6-(CHPh_2_)_2_-4-*^i^*Pr-C_6_H_2_]} **70** prepared by the reduction of the germylene R(Cl)Ge: with [(^Mes^Nacnac)Mg]_2_ [28]. However, given the Ge–Ge bond distance of 2.3568(3) Å in **70** that is markedly longer than those of Power’s di(aryl)digermynes [84,87], it is better formulated as a digermene (WBI = 1.75) [28], rather than a digermyne.

By contrast, the constitution of the stable di(aryl)digermyne Bbt–Ge≡Ge–Bbt (Bbt = 2,6-[(Me_3_Si)_2_CH]_2_-4-[(Me_3_Si)_3_C]-C_6_H_2_) **71**, as the triply bonded derivative, was firmly established by Tokitoh and coworkers [88]. **71** was readily prepared by the reduction of the 1,2-di(bromo)digermene precursor *trans*-[Bbt(Br)Ge=Ge(Bbt)Br] with KC_8_. The two crystallographically independent molecules in the unit cell of **71** showed slightly twisted [C_Ar_–Ge–Ge–C_Ar_ torsional angles = 160.2° and 168.9°] and very short [*r*_Ge≡Ge_ = 2.2060(7) Å and 2.2260(7) Å] Ge–Ge bonds (markedly shorter that those in Power’s digermynes **65** and **67–69**).

Reducing 1,2-di(bromo)digermene (*E*)-Tbb(Br)Ge=Ge(Br)Tbb (Tbb = 2,6-[(Me_3_Si)_2_CH]_2_-4-*^t^*Bu-C_6_H_2_) with KC_8_, Sasamori, Tokitoh and coworkers synthesized novel di(aryl)digermyne Tbb–Ge≡Ge–Tbb **72** [19]. As other digermynes, **72** displayed short Ge≡Ge bond [*r*_Ge≡Ge_ = 2.2410(9)/2.2221(9) Å] and *trans*-bent C_aryl_–Ge–Ge angle (130.5/130.7°) (for two independent molecules in the unit cell of **72**).

### 3.2. Heteronuclear Derivatives

#### Germynes –Ge≡C–

Heteronuclear alkyne analogues of the type –Ge≡E– (E = C, Si, Sn, Pb), featuring a triple bond between germanium and different group 14 element, were always among the top challenges for experimental pursuits. However, despite numerous research efforts, such compounds (as stable derivatives) have not been isolated to date, although germynes R–Ge≡C–R′ were most closely approached, both computationally and experimentally.

Theoretical studies by Su and a coworker predicted that the germynes can be synthetically accessible, if appropriately substituted with bulky groups to prevent isomerization and oligomerization of highly reactive germynes [89].

The first chemical evidence for the transient germyne ArGe≡CSiMe_3_ [Ar = 2,6-(*^i^*Pr_2_NCH_2_)_2_-C_6_H_3_], generated by the low-temperature photolysis (*λ* = 300 nm, −50 C°) of (diazo)germylene precursor ArGe[C(N_2_)SiMe_3_] and trapped with *^t^*BuOH forming ArGe(O*^t^*Bu)_2_CH_2_SiMe_3_, was reported by Couret and coworkers [90]. The transient germyne ArGe≡CSiMe_3_ [Ar = 2,4-*^t^*Bu-6-(*^i^*Pr_2_NCH_2_)-C_6_H_2_] was similarly generated and trapped with water and *^t^*BuOH by Mazières and coworkers [91].

Likewise, photolyzing stable (diazo)germylene, the Kato and Baceiredo group generated metastable (stable below −30 °C) phosphine-stabilized germyne [92]. This compound, however, featured a very long Ge–C separation, [*r*_Ge–C_ = 1.887(5) Å], a value that was intermediate between those of typical Ge–Ge single and Ge=Ge double bonds. Accordingly, it is better described as a singly bonded bis(carbenoid) [:Ge–C:] rather than a triply bonded germyne [Ge≡C].

## 4. Heavy Analogues of 1,3-Dienes

### 4.1. Homonuclear Derivatives

#### Tetra(germa)buta-1,3-dienes >Ge=Ge–Ge=Ge<

**Scheme 31:**
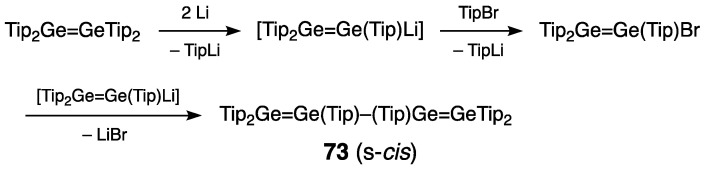
Synthesis of the first isolable tetragermabuta-1,3-diene **73**.

The very first compound of this type, Tip_2_Ge=Ge(Tip)–Ge(Tip)=GeTip_2_ (Tip = 2,4,6-*^i^*Pr_3_-C_6_H_2_) **73**, was synthesized in 2000 by Weidenbruch and coworkers via the reduction of the digermene Tip_2_Ge=GeTip_2_ with lithium, followed by the reaction of the resulting digermenyllithium [Tip_2_Ge=Ge(Tip)Li] with TipBr (Scheme 31) [54]. The Ge–Ge bonds in **73** are in accord with the formulation of the 1,3-diene system >Ge1=Ge2–Ge3=Ge4<, being 2.3568(6) Å (for Ge1=Ge2), 2.4581(5) Å (for Ge2–Ge3), and 2.3439(5) Å (for Ge3=Ge4), respectively. Both Ge=Ge bonds in **73** are *trans*-bent [*θ* = 35.4°/31.1° (for Ge1=Ge2) and 33.3°/31.1° (for Ge3=Ge4)] and twisted [*τ* = 22.4° (Ge1=Ge2) and 21.3° (for Ge3=Ge4)], as is typical for the digermenes. The longest wavelength absorption of **73** in hexane is 560 nm, which remarkably exceeded those of yellow or orange digermenes by 140 nm [and even those of tetra(silyl)digermenes by ca. 100 nm], thus testifying to the presence of conjugation between the two Ge=Ge double bonds.

The same authors later reported improved synthesis of **73** by the one-pot reaction of GeCl_2_∙dioxane complex, TipMgBr, and Mg in THF, allowing for an increase of the isolated yield of **73** to 31% (cf.: 11% [50]) [57]. In *o*-dichlorobenzene, tetra(germa)buta-1,3-diene **73** revealed a reversible reduction [*E*_1/2_(red) = –0.46 V] and irreversible oxidation [*E*_p_(ox) = 0.15 V], whereas in THF both reduction and oxidation processes were irreversible [*E*_p_(red) = –1.75/–1.3 V and *E*_p_(ox) = 0.6 V] [93].

**Scheme 32:**
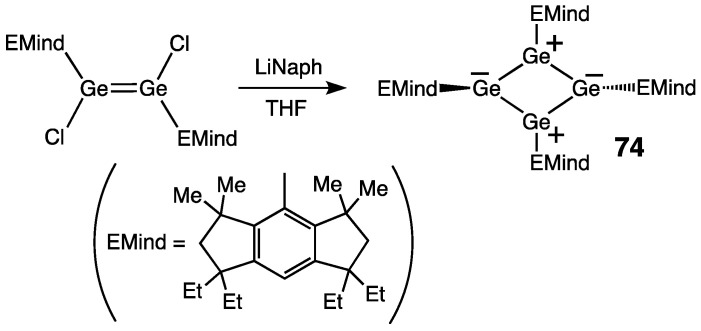
Synthesis of tetra(germa)cyclobutadiene **74**.

Apart from **73**, there is only one tetra(germa)butadiene derivative reported by Matsuo and coworkers, tetra(germa)cyclobutadiene Ge_4_[EMind]_4_ (EMind = 1,1,7,7-tetraethyl-3,3,5,5-tetramethyl-*s*-hydrindacen-4-yl) **74** (as the germanium analogue of the isostructural tetra(sila)cyclobutadiene [94]) [95]. Moreover, **74** was available by the reduction of di(chloro)digermene [EMind](Cl)Ge=Ge(Cl)[EMind] with lithium naphthalenide (Scheme 32). In contrast to tetra(sila)cyclobutadiene, tetra(germa)cyclobutadiene **74** was thermally stable, with the UV-absorptions observed at 458 (ε = 15000), 510 (ε = 7400), and 836 nm (ε = 150). The Ge_4_-ring is rhombic-planar [***Σ***_internal bond angles_ = 360°] with the cyclic Ge–Ge bonds of 2.430 Å (av.). Two germanium atoms feature sp^2^-like trigonal-planar geometry (***Σ***_Ge_ = 360.0°), whereas the other two germanium atoms have sp^3^-like pyramidal configuration (***Σ***_Ge_ = 334.0 and 327.7°) with the *trans*-bending of substituents (*θ* = 37.9 and 40.6°). Theoretical studies revealed that the Ge–Ge bond orders in **74** = 1.08–1.09 (WBI). Within the Ge_4_-ring, there is a clear charge separation: if the two diagonal Ge atoms are slightly positively charged (+0.146 and +0.151, NPA charges), the other two Ge atoms are strongly positively charged (+0.602 and +0.573, NPA charges). All of these data are indicative of the crucial contribution of the charge-separated resonance form *cyclo*-[Ge^+^–Ge^−^–Ge^+^–Ge^−^], which was also the case for the previously reported tetra(sila)cyclobutadiene. The polar Jahn–Teller distortion results in the relaxation of the inherent 4*π*-antiaromaticity of **74**, thus forming a charge-separated rhombic–planar singlet structure, which is nonaromatic (based on the NICS values).

### 4.2. Heteronuclear Derivatives

#### 4.2.1. 1-Sila-2-germabuta-1,3-dienes >Si=Ge–C=C<

**Scheme 33:**
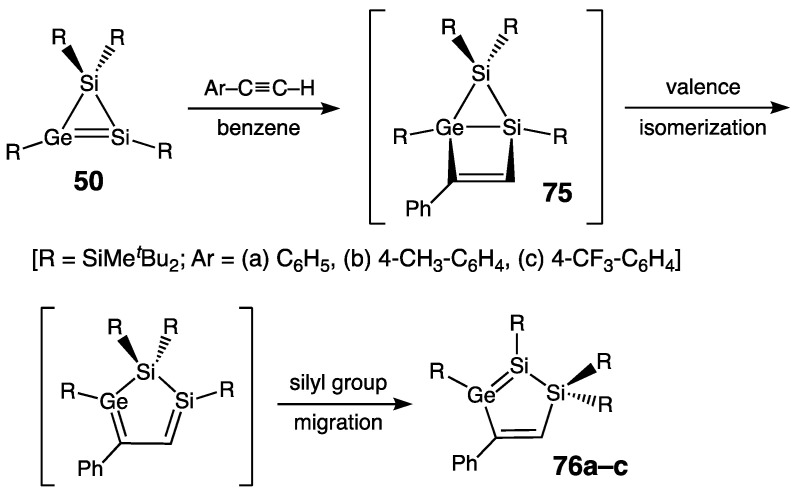
Synthesis of 1,2-disila-3-germacyclopenta-2,4-dienes **76a**–**c** containing Si=Ge–C=C system.

Lee, Sekiguchi, and coworkers developed a different approach toward 1,3-dienes containing Ge atoms. They synthesized five-membered ring derivatives including the >Si=Ge–C=C< 1,3-diene fragment, starting from the [2 + 2] cycloaddition of the heavy cyclopropene (1*H*-disilagermirene **50** [49]) with terminal alkynes to yield bicyclo[2.1.0]pentenes **75**, which underwent valence isomerization, finally forming 1,2-disila-3-germacyclopenta-2,4-dienes **76** [^29^Si NMR (sp^2^-Si): +124.2 ppm (**76a**), +123.6 ppm (**76b**), and +127.3 ppm (**76c**)] (Scheme 33) [96,97,98]. Such heavy cyclopentadienes **76** constitute rather unusual cyclic systems with two formally conjugated double bonds, Si=Ge and C=C, which are actually isolated, despite the planarity of the five-membered ring. This conclusion was based on the crystallographic and UV spectroscopic data. Thus, in the Si=Ge–C=C fragment, all bonds are within the standard ranges (for example, for **76a**, 2.250(1) Å (Si=Ge), 1.972(3) Å (Ge–C), and 1.343(5) Å (C=C)); that is, no elongation of the double bonds and shortening of the single bonds expected for the conjugated system were observed. Likewise, there was no notable red-shift in the longest wavelength UV absorption (*π*–π*) of **76a** compared to that of the starting **50** with isolated Si=Ge bond, 472 vs. 467 nm, respectively. Most likely, the lack of 1,3-conjugation between the Si=Ge and C=C bonds in 1,2-disila-3-germacyclopenta-2,4-dienes **76** results from the substantial spatial and energetic mismatch of the Si=Ge and C=C bonds molecular orbitals, because effective overlap of the latter is a prerequisite for the *π*-electrons delocalization.

#### 4.2.2. 1,2-di(germa)cyclobuta-1,3-diene >Ge=Ge–C=C<

**Scheme 34:**
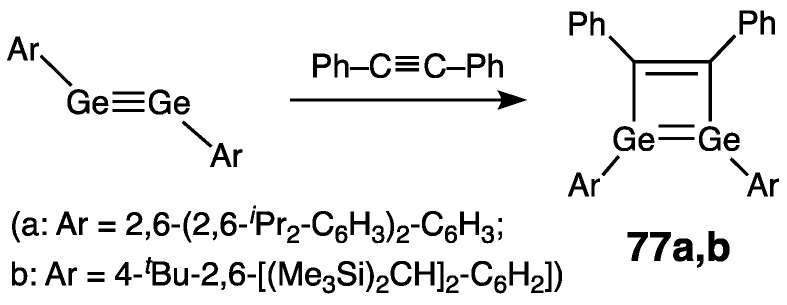
Synthesis of stable 1,2-di(germa)cyclobutadienes **77a**,**b**.

This synthetic strategy toward Ge=Ge–C=C conjugated systems was pioneered by Power’s group and then developed by Sasamori and coworkers, who prepared and isolated stable 1,2-di(germa)cyclobutadienes **77a**,**b** via the reaction of their respective digermynes with diphenylacetylene (Scheme 34) [99,100,101]. In both **77a** and **77b**, the Ge_2_C_2_-ring is practically planar (***Σ***_internal bond angles_ = 359.8° (**77a**) [99] and 359.9° (**77b**) [101]), albeit the Ge centers are strongly pyramidalized [***Σ***_Ge_ = 318.8° and 317.3° (**77a** [99])]. Within the Ge=Ge–C=C fragment, the bonds germanium–germanium/germanium–carbon/carbon–carbon are classified as double/single/double bonds, respectively: 2.4708(9)/[2.022(5) and 2.027(5)]/1.365(7) Å (**77a**) [99], and 2.4160(5)/2.022(2)/1.362(5) Å (**77b**) [101]. Accordingly, the structure of **77a,b** is best described by the resonance form *cyclo*-[Ge=Ge–C=C] with the weak Ge=Ge double bond, as depicted in Scheme 34.

**Scheme 35:**
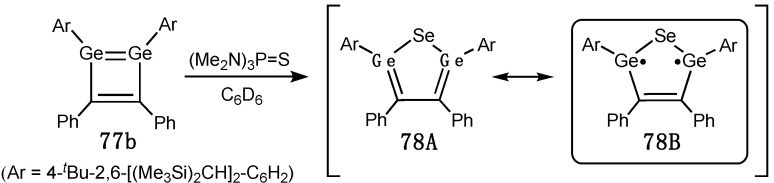
Synthesis of 2,5-di(germa)selenophene **78**.

By reacting 1,2-di(germa)cyclobutadiene **77b** [101] with (Me_2_N)_3_P=Se, Sasamori and coworkers synthesized 2,5-di(germa)selenophene **78**, formed through the transient “housene”-type bicyclic selenadigermirane with its subsequent isomerization to **78** (Scheme 35) [102]. The selenium nucleus in **78** resonated in the low-field region at 481.8 ppm (^77^Se NMR). On the basis of its structural data [*r*_Ge–C_ = 1.921(3)/1.922(3) Å, *r*_C–C_ = 1.375(4) Å, *θ*_Ge_ = 7.8°(*trans*), nonplanar SeGe_2_C_2_-ring] and computational studies, **78** is better formulated as a singlet (digerma)biradicaloid **78B**, while classical Lewis representation **78A** is a minor contributor (Scheme 35). Nevertheless, **78** exhibited some degree of aromaticity, as its NICS(1) of –8.0 was comparable with that of the organic selenophene.

## 5. Heavy Analogues of Allene

### 5.1. Tri(germa)allene >Ge=Ge=Ge<, Germadi(sila)allene >Si=Ge=Si<, and Di(germa)silaallene >Ge=Si=Ge< 

**Scheme 36:**
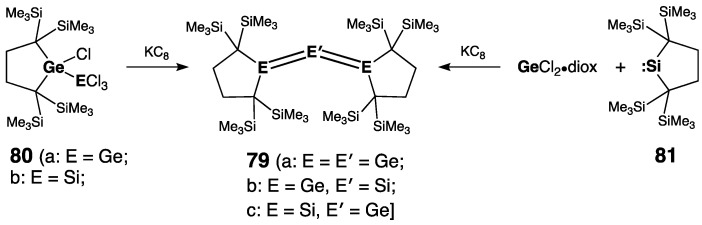
Synthesis of tri(germa)allene **79a**, 1,3-di(germa)silaallene **79b**, and 2-germadi(sila)allene **79c**.

Following their original report on the synthesis of the stable tri(sila)allene >Si=Si=Si< [103], Kira and coworkers subsequently prepared tri(germa)allene **79a** [104], 1,3-di(germa)silaallene **79b** [104], and 2-germadi(sila)allene **79c** [105]. Of them, **79a** and **79b** were prepared by the co-reduction of tetra(chloro)digermane **80a** and chloro(trichlorosilyl)germane **80b** with KC_8_, whereas **79c** was synthesized by the co-reduction of di(alkyl)silylene **81** and GeCl_2_∙diox with KC_8_ (Scheme 36). All sp^2^-Si atoms were diagnostically deshielded, with the central Si atom in **79b** being more strongly deshielded than the peripheral Si atoms in **79c**: +236.6 ppm vs. +219.4 ppm. The longest wavelength UV absorptions in **79a**, **79b**, and **79c** were found at 630, 612, and 599 nm, respectively, thus pointing to the significant conjugation between the cumulated double bonds. The allenic bonds in **79** were all in the range of the typical double bonds: *r*_Ge=Ge_ = 2.321(2) Å and 2.330(3) Å in **79a**, *r*_Ge=Si_ = 2.2697(8) Å in **79b**, and *r*_Si=Ge_ = 2.2366(7) Å and 2.2373(7) Å in **79c**. The allenic E=E′=E framework in **79** was notably bent [bending angle = 122.61(6)° (in **79a**), 125.71(7)° (in **79b**), and 132.38(2)° (in **79c**)], and the terminal atoms E were strongly pyramidalized [*θ*_E_ = 348.5°/348.6° (in **79a**), 349.3° (in **79b**), and 353.9°/354.0° (in **79c**)].

**Scheme 37:**
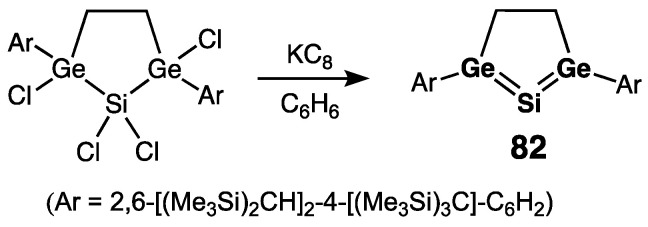
Synthesis of cyclic 1,3-di(germa)-2-silaallene **82**.

Apart from Kira’s heavy allenes described above, there was only one recent report on the title compounds: Sasamori, Tokitoh, and coworkers prepared cyclic 1,3-di(germa)-2-silaallene **82** via the reductive dechlorination of tetra(chloro)-2,5-digerma-1-silacyclopentane with KC_8_ (Scheme 37) [106]. In **82**, the SiGe_2_C_2_-ring is planar, and the germanium–silicon bonds are typical double bonds [*r*_Si=Ge_ = 2.2681(18) and 2.2900(18) Å]. However, the central allenic Si in **82** was extraordinarily shielded [*δ*(^29^Si) = –16.5 ppm] [106] compared to that of Kira’s 1,3-di(germa)-2-silallene (236.6 ppm) [104]. This was explained by the contribution of the “silylone” resonance structure [>Ge:→Si^0^←:Ge<] of **82**, resulting from the acute Ge–Si–Ge bond angle (80.1°) due to incorporation of the Ge–Si–Ge unit into the five-membered ring skeleton. This was supported by calculations which showed that the germanium–silicon bonds in **82** are highly polarized as Ge*^δ^*^+^–Si*^δ^*^−^, –0.27 for Si and +0.90/+0.91 for Ge (NPA charges), and this is indicative of an important Ge-to-Si *σ*-donation.

### 5.2. Germaallenes >Ge=C=C<

Only a couple of isolable 1-germaallenes >Ge=C=C< are currently known. The first one, Tip_2_Ge=C=C(*^t^*Bu)Ph **83**, was reported by West and coworkers synthesized by the reaction of Tip_2_Ge(F)–C≡C–Ph with *^t^*BuLi via the transient [Tip_2_Ge(F)–C(Li)=C(*^t^*Bu)Ph], which eliminated LiF to finally yield **5** [107]. In solution, **83** decomposed at room temperature within 15 h. In **83**, as in other heteroallenes, the Ge=C and C=C bonds were short [*r*_Ge=C_ = 1.783(2) Å and *r*_C=C_ = 1.314(2) Å], the Ge=C=C fragment was bent (159.2°), the geometry at the Ge atom was pyramidal (***Σ***_Ge_ = 348.4°), and the central C atom was greatly deshielded [*δ*(^13^C) = +235.1 ppm].

The second 1-germaallene, Tbt(Mes)Ge=C=CR_2_ (CR_2_ = fluorenylidene) **84**, was prepared by the reductive dechlorination of Tbt(Mes)ClGe–C(Cl)=CR_2_ with *^t^*BuLi [108]. Like in **83**, the central allenic carbon of **84** resonated in the diagnostic low-field at +243.6 ppm. Without trapping reagents, **84** underwent slow intramolecular cyclization via C–H activation of one of the (Me_3_Si)_2_CH groups by the Ge=C bond to form a benzogermacyclobutene derivative.

## 6. Heavy Analogues of Vinylidenes

Vinylidenes R_2_C=C:, with substituent-free terminal carbon atoms, are the valence isomers of alkynes RC≡CR. They are exceptionally reactive, and for their stabilization, external bases, such as NHC, are particularly effective. Accordingly, all—except for Aldridge’s di(germa)vinylidene (vide infra)—isolable heavy group 14 analogues of vinylidenes R_2_E=E′: (E, E′ = heavy group 14 elements) were stabilized by NHC-coordination.

### 6.1. Homonuclear Derivatives

#### Di(germa)vinylidene >Ge=Ge:

**Scheme 38:**
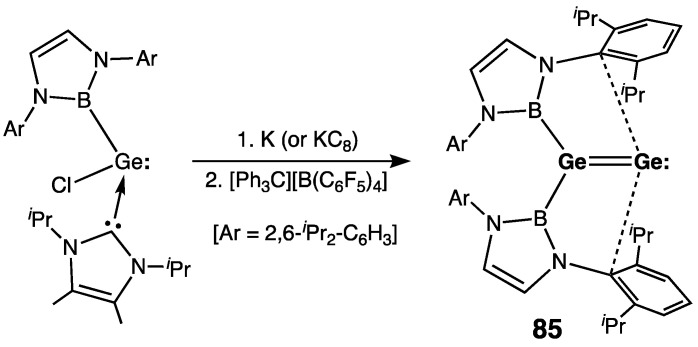
Synthesis of the first digermavinylidene **85**.

The first di(germa)vinylidene **85**, synthesized by Aldridge and coworkers, was free from external NHC-coordination, in contrast to the NHC-supported silagermenylidenes (vide infra) (Scheme 38) [109].

**85** was prepared by the reduction of the boryl(chloro)germylene–NHC complex with K (or KC_8_), followed by the oxidation of the intermediate dianion by [Ph_3_C]^+^[B(C_6_F_5_)_4_]^−^ (Scheme 38). The germanium–germanium bond in **85** is planar and short [*r*_Ge=Ge_ = 2.312(1) Å] in order to formulate it as a Ge=Ge double bond. In the crystal, weakly stabilizing interactions were found between one of the aryl *π*-systems of each of the boryl substituents and the vacant *p*-orbital at the monocoordinated Ge atom. The HOMO of **85** is the Ge=Ge *π*-bond, whereas the HOMO–1 is mostly the naked Ge lone pair. The longest wavelength UV-absorption in **85**, observed at 460 nm, is due to the Ge=Ge bond *π*–π* electronic transition.

**Scheme 39:**
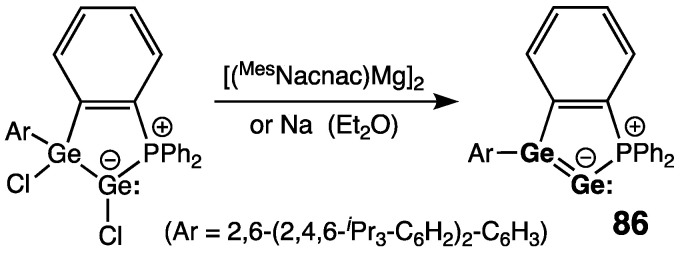
Synthesis of digermavinylidene **86**.

Another stable di(germa)vinylidene **86** was reported by Wesemann and coworkers, synthesized by the reduction of intramolecularly phosphine-stabilized chloro(germyl)germylene with [(^Mes^Nacnac)Mg]_2_ (^Mes^Nacnac = {[(2,4,6-Me_3_-C_6_H_2_)N–(Me)C]_2_CH}^−^) or metallic sodium (Scheme 39) [110]. The germanium–germanium bond in **86** [*r*_Ge=Ge_ = 2.3060(2) Å] is within the range of the Ge=Ge double bonds, but it is slightly shorter than that in **85** [*r*_Ge=Ge_ = 2.312(1) Å]; the bicyclic framework is planar, and the tricoordinate sp^2^-Ge disclosed nearly planar geometry (***Σ***_Ge_ = 358.8°). As the manifestation of the Ge=Ge double bonding in **86**, *π*_Ge=Ge_ and π*_Ge=Ge_ orbitals represent the HOMO and LUMO, respectively. The bonding situation in **86** can be alternatively viewed as a double Lewis base-stabilized Ge atom in the formal oxidation state 0. Thus, di(germa)vinylidene **86** can potentially serve as a source for Ge^0^-single atom transfer by the germanium abstraction reactions.

### 6.2. Heteronuclear Derivatives 

#### 6.2.1. Silagermenylidene >Si=Ge:

The first isolable heavy Group 14 elements analogue of the vinylidene, NHC-stabilized silagermenylidene Tip_2_Si=Ge:(⟵NHC) (NHC = 1,3-diisopropyl-4,5-dimethylimidazol-2-ylidene) **87**, was prepared by the co-reduction of Tip_2_SiCl_2_ and [NHC→GeCl_2_] complex with lithium naphthalenide, as reported by Scheschkewitz and coworkers [111]. As is typical for the Si=Ge doubly bonded system, the Si atom in **87** is markedly deshielded [*δ*(^29^Si) = 158.9 ppm] and has a planar configuration (***Σ***_Si_ = 359.8°), and the Si=Ge bond is short [*r*_Si=Ge_ = 2.2521(5) Å]. Moreover, the Si=Ge bond in **87** is practically untwisted (*τ* = 3.1°), which favors an effective *p*_π_–*p*_π_ orbital overlap, and the NHC-ligand coordinates to the Ge nearly orthogonally (C_NHC_–Ge–Si bond angle = 98.9°), thus maximizing the [*n*_NHC lone pair_ → *p*_Ge_] orbital interaction. Subsequently, the same group prepared another NHC-stabilized silagermenylidene, [Tip_2_(Cl)Si](Tip)Si=Ge:(⟵NHC) (NHC = 1,3-diisopropyl-4,5-dimethylimidazol-2-ylidene) **88**, whose spectral and structural characteristics are similar to those of **87**: *δ* (^29^Si) = 162.5 ppm, *r*_Si=Ge_ = 2.2757(10) Å, C_NHC_–Ge–Si bond angle = 101.9° [112].

## 7. Digermanium(0) Complexes :Ge=Ge:

**Scheme 40:**
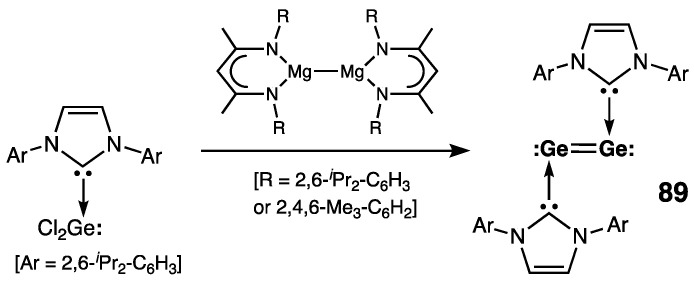
Synthesis of the digermanium(0) NHC-complex **89**.

The new class of low-coordinate organogermanium compounds with the central Ge atom in zero formal oxidation state emerged recently. This was preceded by the synthesis of the disilicon analogue [NHC→**Si^0^=Si^0^**←NHC] (NHC = 1,3-(2,6-*^i^*Pr_2_-C_6_H_3_)_2_-imidazol-2-ylidene, which was reported by Robinson and coworkers in 2008 [113]. Following this original report, Jones and coworkers synthesized digermanium(0) complex [NHC→**Ge^0^=Ge^0^**←NHC] (NHC = 1,3-(2,6-*^i^*Pr_2_-C_6_H_3_)_2_-imidazol-2-ylidene) **89** [114] and ditin(0) complex [NHC→**Sn^0^=Sn^0^**←NHC] (NHC = 1,3-(2,6-*^i^*Pr_2_-C_6_H_3_)_2_-imidazol-2-ylidene) [115]. Moreover, **89** was prepared by the reduction of the di(chloro)germylene–NHC complex with *β*-diketiminate magnesium(I) reagents [{Mg([N(R)CMe]_2_CH)}_2_] (R = 2,6-*^i^*Pr_2_-C_6_H_3_ or 2,4,6-Me_3_-C_6_H_2_) (Scheme 40) [114]. Like its silicon analogue [NHC→**Si^0^=Si^0^**←NHC] [113], **89** revealed a characteristically short Ge=Ge bond [*r*_Ge=Ge_ = 2.3490(8) Å] and orthogonal coordination of the NHC ligands to the Ge=Ge unit (C–Ge–Ge bond angle = 89.9°). Accordingly, **89** was classified as a singlet digermanium(0) derivative, :Ge=Ge:, datively stabilized by the NHC ligands’ coordination.

**Scheme 41:**
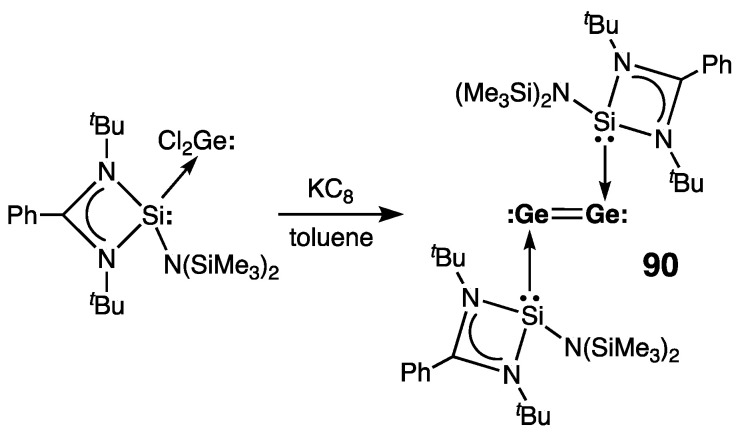
Synthesis of digermanium(0) NHSi-complex **90**.

As the latest development in the field, So and coworkers reported *N*-heterocyclic silylene(NHSi)-stabilized digermanium(0) complex **90**, which was prepared by the reduction of NHSi–GeCl_2_ complex with KC_8_ (Scheme 41) [116]. In **90**, both NHSi-ligands are nearly orthogonal to the [:Ge^0^=Ge^0^:] unit (Si–Ge–Ge bond angle = 92.0°), and the Ge=Ge bond [*r*_Ge=Ge_ = 2.3518(16) Å] is still short enough to be in the range of the typical Ge=Ge bonds in digermenes. However, in contrast to digermanium(0) complex **89** [114], **90** revealed *π*-back-bonding from the Ge=Ge *π*-bond to the vacant *p*(Si)-orbital of the NHSi ligand which stabilizes the whole molecule, as was shown by the frontier MO and AIM analyses.

## 8. Prospects for the Use of Unsaturated Organogermanium Compounds

Organogermanium compounds featuring multiple bonds to germanium are highly promising candidates for their potential use in synthetic organic and organometallic chemistry, as well as in the materials science field, due to the presence of highly reactive germanium-containing double and triple bonds. Although there is still much to be achieved in this direction, some prominent examples of the synthetic utility of unsaturated organogermanium compounds have been reported to date, mostly for digermenes and digermynes, as the most abundant representatives of the class.

**Scheme 42:**
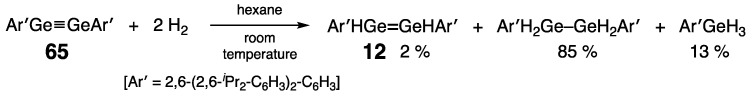
Activation of dihydrogen by digermyne **65**.

Among the most important examples of digermynes reactivity, one can mention their activation of small molecules (H_2_, NH_3_, CO, CO_2_, N_2_O, etc.), which typically can be activated under the transition metal catalysis conditions. Thus, in 2005, Power and coworkers reported facile room temperature/atmospheric pressure activation of dihydrogen by digermyne Ar′Ge≡GeAr′ [Ar′ = 2,6-(2,6-*^i^*Pr_2_-C_6_H_3_)_2_-C_6_H_3_] **65** as the first example of H_2_ addition to an unsaturated main group compound under mild non-catalytic conditions (Scheme 42) [25]. This reaction, producing a mixture of doubly bonded digermene Ar′HGe=GeHAr′ **12**, singly bonded digermane Ar′H_2_Ge–GeH_2_Ar′, and primary germane Ar′GeH_3_, depending on the amount of H_2_ gas used, is likely enabled by the highly reactive singlet biradical contribution of the germanium–germanium bonding.

**Scheme 43:**
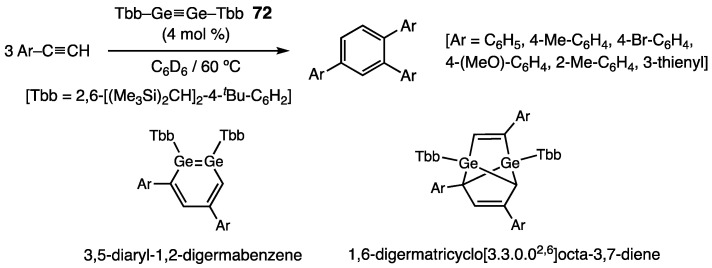
Regioselective cyclotrimerization of terminal arylacetylenes catalyzed by digermyne **72**.

An example of the catalytic application of a digermyne was recently reported by Sasamori and coworkers. By applying catalytic amounts (4 mol%) of their di(aryl)digermyne Tbb–Ge≡Ge–Tbb (Tbb = 2,6-[(Me_3_Si)_2_CH]_2_-4-*^t^*Bu-C_6_H_2_) **72** [19], the authors achieved a high-yield regioselective cyclotrimerization of terminal arylacetylenes Ar–C≡CH, exclusively forming 1,2,4-triarylbenzenes (Scheme 43) [117]. Digermyne **72** acts as a highly efficient pre-catalyst of the process [turnover number (TON) = 15–35], which can be regarded as the transition metal-free variation of the Reppe reaction that is catalyzed by the main group element derivative instead of by transition metals (Mo, Co). The mechanism of the whole catalytic transformation was backed by control experiments and accompanying computations, which revealed initial reaction of digermyne **72** with two equivalents of arylacetylene generating 3,5-diaryl-1,2-digermabenzene intermediate, followed by its reaction with another equivalent of arylacetylene forming 1,6-digermatricyclo[3.3.0.0^2,6^]octa-3,7-diene as a key resting state for the true catalytic species (Scheme 43). Readily switching between Ge^II^ and Ge^IV^ oxidation states, mimicking the fundamentally important switching between oxidation states in the transition metal catalytic processes, finally enables smooth cyclotrimerization of arylacetylenes.

**Scheme 44:**
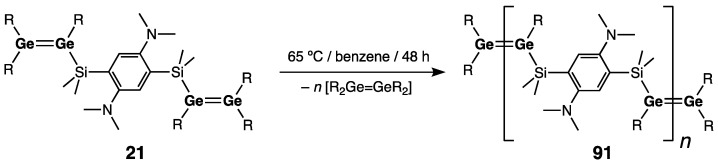
The acyclic diene metathesis (ADMET) polymerization of *p*-phenylene-bridged tetragermadiene precursor **21** forming ADMET-poly(digermene) **91**.

As was described in Section 2.1.1 (Acyclic digermenes), very recently Scheschkewitz and coworkers developed an interesting germanium version of ADMET polymerization by the thermolysis of *p*-phenylene-bridged digermene-terminated tetragermadiene precursor **21** to form ADMET-poly(digermene) **91** (number-average degree of polymerization = 23, mass-average degree of polymerization = 45, and dispersity index = 1.95) (Scheme 44) [40].

## 9. Conclusions

The field of compounds with multiple bonds to germanium, i.e., *organogermanium analogues of alkenes, alkynes, 1,3-dienes, allenes, and vinylidenes*, established by the synthesis of the first stable digermene, Dis_2_Ge=GeDis_2_, by Lappert and coworkers in 1976, is thriving. Currently, a huge variety of the above-described unsaturated organogermanium derivatives have been prepared and, in the majority of cases, structurally characterized. These compounds are no longer short-lived reaction intermediates; instead, they are commonly available preparative-scale reagents indispensable for the synthesis of organogermanium derivatives unavailable by any other synthetic route. The fundamentally important issue of the particular structure and nature of Ge=E double and Ge≡E triple bonds (E = group 14 element), as compared with those in the prototypical organic alkenes C=C and alkynes C≡C, was comprehensively addressed (both experimentally and theoretically) to show the principal distinctions in their bonding types: **classical covalent** in the planar alkenes C=C and alkynes C≡C vs. **nonclassical donor–acceptor** in the *trans*-bent organogermanium analogues Ge=E and Ge≡E. For future developments in the field, one can expect the successful design of still unknown types of multiple (both double and triple) bonding between Ge and the Main Group elements, such as R–Ge≡E–R′ (E = Si, Sn, Pb), which are among the most desirable candidates for experimental pursuits. Among the practical applications of unsaturated organogermanium compounds in material science, one can mention the potential use of germaalkenes >Ge=E< and germaalkynes –Ge≡E– (E = group 14 element) as novel ligands for transition metal complexes of the next generation, as well as the development of the germanium variation of alkene metathesis for the fabrication of Ge-containing polymers with predesigned properties.

## Figures and Tables

**Table 1:** X-ray crystallographic parameters of the stable acyclic digermenes (reported after 2000). The“**—**” implies the lack of the relevant structural information.

Digermene (Preparation Method)	*r*_Ge=Ge_ (Å)	*θ* (°)	*Σ*_Ge_ (°)	*τ*_Ge=Ge_ (°)	Reference
**1** (B)	2.347(2)	32.0	348.5	0.0	[12]
**2** (A)	2.2856(8)	33.4	348.1	2.9	[14]
**3a** (B)	2.3173(3)	39.7	342.9	—	[15]
**3b** (B)	2.347(3)	37.9	343.0	—	[15]
**3c** (B)	2.3183(5)	33.7	348.4	—	[15]
**4** (B)	2.3224(4)	41.7	341.9	—	[16]
**5** (B)	2.3643(4)	37.2/42.6	—	—	[17]
**6** (B)	2.5087(7)	44.6	—	—	[18]
**7** (B)	2.4065(6)/ 2.3970(6)	—	335.5/336.3	—	[19]
**9** (C)	2.2703(8)	0.3	360	7.5	[22]
**10** (C)	2.416(2)	—	356.6/355.3	16.5/34.6	[23]
**11** (C)	2.3320(5)	43.7	337.8	—	[24]
**12** (D)	2.3026(3)	45.0	—	—	[25]
**13** (D)	2.3098(5)	36.8 [GeAr′R]	345.3 [GeAr′R]	—	[27]
**14** (C)	2.346(2)	—	358.8/359.2	52.8	[31,32]
**15** (special)	2.2944(4)	37.7	334.5	—	[34]
**16** (special)	2.284(6)	7.1/12.8	—	19.9	[35]
**17a** (special)	2.4119(5)	44.3	335.9	—	[37]
**17b** (special)	2.4145(3)	43.3	337.1	—	[37]
**18** (B)	2.300(2)	—	353.1/353.6	—	[38]
**19** (B)	2.380(1)	—	—	—	[39]
**20** (special)	2.2576(5)	21.5	—	0	[40]
**21** (special)	2.3038(4)	24.9/31.9	—	18.0	[40]

**Table 2:** X-ray crystallographic parameters of the stable cyclic digermenes. The “**—**” implies lack of the relevant structural information.

Cyclic Digermene	*r*_Ge=Ge_ (Å)	*Σ*_Ge_ (°)	*τ*_Ge=Ge_ (°)	Ring Folding (°)	Reference
*Heavy cyclopropenes*					
**22a**	2.239(4)	359.9	—		[41]
**23c**	2.264(2)	358.7/359.8	8.1		[43]
**24a**	2.2723(8)	357.2/359.3	29.7		[44]
**24b**	2.2743(8)	343.7/359.4	34.9		[44]
**24c**	2.2721(6)	344.8/359.6	35.3		[44]
**25a**	2.2429(6)	353.8/354.2	51.0		[45,46]
*Heavy cyclobutenes*					
**27**	2.2911(4)	357.2/358.6	—	28.3	[50]
**28**	2.2993(5)	354.4/356.4	—	24.4/24.6	[51]
**29**	2.2808(7)	—	—	—	[52]
**30**	2.4132(5)	—	—	11.1	[53]
*Heavy cyclopentenes*					
**36a**	2.2841(5)	348.4/351.4	—	—	[57]
**36b**	2.2975(5)	349.2/349.9	—	—	[52]
**37**	2.2663(9)	360.0/360.0	16.2	—	[58]
*Heavy cyclohexene*					
**38**	2.2896(6)	359.5/360.0	5.2	—	[58]

## Data Availability

Not applicable.

## Abbreviations

ADMET	Acyclic Diene METathesis
AIM	Atoms in Molecules
B3LYP	Becke Three-Parameter Hybrid Functional with the Lee–Yang–Parr Correlation Functional
Bbt	2,6-[(Me_3_Si)_2_CH]_2_-4-[(Me_3_Si)_3_C]-C_6_H_2_
cc-pVDZ-pp	Correlation-Consistent Polarized Valence Double Zeta with Pseudo Potential Basis Set
CV	Cyclic Voltammetry
diox	1,4-dioxane
Dis	(Me_3_Si)_2_CH
Eind	1,1,3,3,5,5,7,7-octaethyl-*s*-hydrindacen-4-yl
EMind	1,1,7,7-tetraethyl-3,3,5,5-tetramethyl-*s*-hydrindacen-4-yl
EPR	Electronic Paramagnetic Resonance
Fc	ferrocenyl
hffc	hyperfine coupling constant
HOMO	Highest Occupied Molecular Orbital
IDipp (or IPr)	1,3-bis(2,6-diisopropylphenyl)-2*H*-imidazol-2-ylidene
LUMO	lowest unoccupied molecular orbital
Mes	2,4,6-Me_3_-C_6_H_2_
MO	Molecular Orbital
NHC	*N*-Heterocyclic Carbene
NHSi	*N*-Heterocyclic Silylene
NICS	Nucleus Independent Chemical Shift
NMR	Nuclear Magnetic Resonance
NPA	Natural Population Analysis
Tbb	2,6-[(Me_3_Si)_2_CH]_2_-4-*^t^*Bu-C_6_H_2_
Tbt	2,4,6-[(Me_3_Si)_2_CH]_3_-C_6_H_2_]
THF	tetrahydrofuran
Tip	2,4,6-*^i^*Pr_3_-C_6_H_2_
UV	ultraviolet
WBI	Wiberg Bond Index

## References

[1] Power P.P. (1999). π-Bonding and the lone pair effect in multiple bonds between heavier main group elements. Chem. Rev..

[2] Tokitoh N., Okazaki R., Rappoport Z. (2002). Multiply bonded germanium, tin and lead compounds. The Chemistry of Organic Germanium, Tin and Lead Compounds.

[3] Power P.P. (2003). Silicon, germanium, tin and lead analogues of acetylenes. Chem. Commun..

[4] Fischer R.C., Power P.P. (2010). π-Bonding and the lone pair effect in multiple bonds involving heavier main group elements: Developments in the new millennium. Chem. Rev..

[5] Lee V.Y., Sekiguchi A. (2010). Organometallic Compounds of Low-Coordinate Si, Ge, Sn and Pb: From Phantom Species to Stable Compounds.

[6] Lee V.Y., Sekiguchi A., Reedijk J., Poeppelmeier K.R. (2013). Multiply bonded compounds of the heavy group 14 elements. Comprehensive Inorganic Chemistry II..

[7] Lee V.Y., Lee V.Y. (2023). Multiple bonds to germanium. Organogermanium Compounds (Theory, Experiment, and Applications).

[8] Goldberg D.E., Harris D.H., Lappert M.F., Thomas K.M. (1976). A new synthesis of divalent group 4B alkyls M[CH(SiMe_3_)_2_]_2_ (M = Ge or Sn), and the crystal and molecular structure of the tin compound. J. Chem. Soc., Chem. Commun..

[9] Wedler H.B., Wendelboe P., Power P.P. (2018). Second-order Jahn–Teller (SOJT) structural distortions in multiply bonded higher main group compounds. Organometallics.

[10] Richards A.F., Brynda M., Power P.P. (2004). Effects of the alkali metal counter ions on the germanium–germanium double bond length in a heavier group 14 element ethenide salt. Chem. Commun..

[11] Davidson P.J., Harris D.H., Lappert M.F. (1976). Subvalent group 4B metal alkyl and amides. Part I. The synthesis and physical properties of kinetically stable bis[bis(trimethylsilyl)methyl]-germanium(II), -tin(II), and -lead(II). J. Chem. Soc., Dalton Trans..

[12] Goldberg D.E., Hitchcock P.B., Lappert M.F., Thomas K.M., Thorne A.J., Fjeldberg T., Haaland A., Schilling B.E.R. (1986). Subvalent group 4B metal alkyl and amides. Part 9. Germanium and tin alkene analogues, the dimetallenes M_2_R_4_ [M = Ge or Sn, R = CH(SiMe_3_)_2_]: X-ray structures, molecular orbital calculations for M_2_H_4_, and trends in the series M_2_R′_4_ [M = C, Si, Ge, or Sn; R′ = R, Ph, C_6_H_2_Me_3_-2,4,6, or C_6_H_3_Et_2_-2,6]. J. Chem. Soc. Dalton Trans..

[13] Snow J.T., Murakami S., Masamune S., Williams D.J. (1984). Synthesis and characterization of tetrakis(2,6-diethylphenyl)digermene. Tetrahedron Lett..

[14] Hurni K.L., Rupar P.A., Payne N.C., Baines K.M. (2007). On the synthesis, structure, and reactivity of tetramesityldigermene. Organometallics.

[15] Stender M., Pu L., Power P.P. (2001). Stabilized terphenyl-substituted digermene derivatives of simple organic groups and their halide precursors: Preference for symmetrically bonded structures. Organometallics.

[16] Lei H., Fettinger J.C., Power P.P. (2010). Synthesis and structures of low-valent alkynyl tin and germanium complexes supported by terphenyl ligands: Heavier group 14 element enediyne analogues. Organometallics.

[17] Pampuch B., Saak W., Weidenbruch M. (2006). New compounds with Ge/Ge and Ge/C multiple bonds. J. Organomet. Chem..

[18] Sasamori T., Sugiyama Y., Takeda N., Tokitoh N. (2005). Structure and properties of an overcrowded 1,2-dibromodigermene. Organometallics.

[19] Sasamori T., Sugahara T., Agou T., Guo J.-D., Nagase S., Streubel R., Tokitoh N. (2015). Synthesis and characterization of a 1,2-digermabenzene. Organometallics.

[20] Al-Rafia S.M.I., Momeni M.R., Ferguson M.J., McDonald R., Brown A., Rivard E. (2013). Stable complexes of parent digermene: An inorganic analogue of ethylene. Organometallics.

[21] Kira M., Iwamoto T., Maruyama T., Kabuto C., Sakurai H. (1996). Tetrakis(trialkylsilyl)digermenes. Salient effects of trialkylsilyl substituents on planarity around the Ge=Ge Bond and remarkable thermochromism. Organometallics.

[22] Iwamoto T., Okita J., Yoshida N., Kira M. (2010). Structure and reactions of an isolable Ge=Si doubly bonded compound, tetra(*t*-butyldimethylsilyl)germasilene. Silicon.

[23] Tokitoh N., Kishikawa K., Okazaki R., Sasamori T., Nakata N., Takeda N. (2002). Synthesis and characterization of an extremely hindered tetraaryl-substituted digermene and its unique properties in the solid state and in solution. Polyhedron.

[24] Sasamori T., Miyamoto H., Sakai H., Furukawa Y., Tokitoh N. (2012). 1,2-Bis(ferrocenyl)digermene: A d–*π* electron system containing a Ge=Ge unit. Organometallics.

[25] Spikes G.H., Fettinger J.C., Power P.P. (2005). Facile activation of dihydrogen by an unsaturated heavier main group compound. J. Am. Chem. Soc..

[26] Richards A.F., Phillips A.D., Olmstead M.M., Power P.P. (2003). Isomeric forms of divalent heavier group 14 element hydrides: Characterization of Ar′(H)GeGe(H)Ar′ and Ar′(H)_2_GeGeAr′•PMe_3_ (Ar′ = C_6_H_3_-2,6-Dipp_2_; Dipp = C_6_H_3_-2,6-Pr*^i^*_2_). J. Am. Chem. Soc..

[27] Summerscales O.T., Caputo C.A., Knapp C.E., Fettinger J.C., Power P.P. (2012). The role of group 14 element hydrides in the activation of C–H bonds in cyclic olefins. J. Am. Chem. Soc..

[28] Hadlington T.J., Hermann M., Li J., Frenking G., Jones C. (2013). Activation of H_2_ by a multiply bonded amido-digermyne: Evidence for the formation of a hydrido-germylene. Angew. Chem. Int. Ed..

[29] Hadlington T.J., Li J., Hermann M., Davey A., Frenking G., Jones C. (2015). Reactivity of amido-digermynes, LGeGeL (L = bulky amide), toward olefins and related molecules: Facile reduction, C–H activation, and reversible cycloaddition of unsaturated substrates. Organometallics.

[30] Kelly J.A., Juckel M., Hadlington T.J., Fernández I., Frenking G., Jones C. (2019). Synthesis and reactivity studies of amido-substituted germanium(I)/tin(I) dimers and clusters. Chem. Eur. J..

[31] Lee V.Y., McNiece K., Ito Y., Sekiguchi A. (2011). A blue digermene (*t*-Bu_2_MeSi)_2_Ge=Ge(SiMe*t*-Bu_2_)_2_. Chem. Commun..

[32] Lee V.Y., McNiece K., Ito Y., Sekiguchi A., Geinik N., Becker J.Y. (2014). Tetrakis(di-*tert*-butylmethylsilyl)digermene: Synthesis, structure, electrochemical properties, and reactivity. Heteroatom Chem..

[33] Aysin R.R., Bukalov S.S., Leites L.A., Lee V.Y., Sekiguchi A. (2019). Electronic structure and conformational isomerism of the digermene (*t*Bu_2_MeSi)_2_Ge=Ge(SiMe*t*-Bu_2_)_2_ as studied by temperature-dependent Raman and UV–vis spectra and quantum-chemistry calculations. J. Organomet. Chem..

[34] Jana A., Huch V., Rzepa H.S., Scheschkewitz D. (2015). A Multiply functionalized base-coordinated Ge^II^ compound and its reversible dimerization to the digermene. Angew. Chem. Int. Ed..

[35] Nieder D., Klemmer L., Kaiser Y., Huch V., Scheschkewitz D. (2018). Isolation and reactivity of a digerma analogue of vinyllithiums: A lithium digermenide. Organometallics.

[36] Klemmer L., Kaiser Y., Huch V., Zimmer M., Scheschkewitz D. (2019). Persistent digermenes with acyl and α-chlorosilyl functionalities. Chem. Eur. J..

[37] Hayakawa N., Sugahara T., Numata Y., Kawaai H., Yamatani K., Nishimura S., Goda S., Suzuki Y., Tanikawa T., Nakai H. (2018). 1,2-Dihalodigermenes bearing bulky Eind groups: Synthesis, characterization, and conversion to halogermylenoids. Dalton Trans..

[38] Mangan R.J., Rit A., Sindlinger C.P., Tirfoin R., Campos J., Hicks J., Christensen K.E., Niu H., Aldridge S. (2020). Activation of protic, hydridic and apolar E–H bonds by a boryl-substituted Ge^II^ cation. Chem. Eur. J..

[39] Mangan R.J., Davies A.R., Hicks J., Sindlinger C.P., Thompson A.L., Aldridge S. (2021). Synthesis, structure and reactivity of terphenyl-substituted germylium-ylidene cations. Polyhedron.

[40] Klemmer L., Thömmes A.-L., Zimmer M., Huch V., Morgenstern B., Scheschkewitz D. (2021). Metathesis of Ge=Ge double bonds. Nature Chem..

[41] Sekiguchi A., Yamazaki H., Kabuto C., Sakurai H., Nagase S. (1995). Cyclotrigermenes: A new unsaturated ring system. J. Am. Chem. Soc..

[42] Sekiguchi A., Tsukamoto M., Ichinohe M. (1997). A Free Cyclotrigermenium cation with a 2π-electron system. Science.

[43] Sekiguchi A., Fukaya N., Ichinohe M., Takagi N., Nagase S. (1999). Synthesis of unsymmetrically substituted cyclotrigermenes and the first example of cis configuration around the Ge=Ge double Bond. J. Am. Chem. Soc..

[44] Sekiguchi A., Ishida Y., Fukaya N., Ichinohe M., Takagi N., Nagase S. (2002). The first halogen-substituted cyclotrigermenes: A unique halogen walk over the three-membered ring skeleton and facial stereoselectivity in the Diels-Alder reaction. J. Am. Chem. Soc..

[45] Lee V.Y., Yasuda H., Ichinohe M., Sekiguchi A. (2005). SiGe_2_ and Ge_3_: Cyclic digermenes that undergo unexpected ring-expansion reactions. Angew. Chem. Int. Ed..

[46] Lee V.Y., Yasuda H., Ichinohe M., Sekiguchi A. (2007). Heavy cyclopropene analogues R_4_SiGe_2_ and R_4_Ge_3_ (R = SiMe*^t^*Bu_2_)—New members of the cyclic digermenes family. J. Organomet. Chem..

[47] McNeice K., Lee V.Y., Sekiguchi A. (2011). Making a cyclotrigermene from a digermene. Organometallics.

[48] Lee V.Y., Yasuda H., Sekiguchi A. (2007). Interplay of E*_n_*E′*_3–n_*C valence isomers (E, E′ = Si, Ge): Bicyclo[1.1.0]butanes with very short bridging bonds and their isomerization to alkyl-substituted cyclopropenes. J. Am. Chem. Soc..

[49] Lee V.Y., Ichinohe M., Sekiguchi A., Takagi N., Nagase S. (2000). The first three-membered unsaturated rings consisting of different heavier group 14 elements: 1-disilagermirene with a Si=Si double bond and its isomerization to a 2-disilagermirene with a Si=Ge double bond. J. Am. Chem. Soc..

[50] Lee V.Y., Takanashi K., Ichinohe M., Sekiguchi A. (2003). A chemical trick: How to make a digermene from a disilene, formation of ^3^Δ-1,2,3,4-disiladigermetene. J. Am. Chem. Soc..

[51] Lee V.Y., Ito Y., Yasuda H., Takanashi K., Sekiguchi A. (2011). From tetragermacyclobutene to tetragermacyclobutadiene dianion to tetragermacyclobutadiene transition metal complexes. J. Am. Chem. Soc..

[52] Ramaker G., Saak W., Haase D., Weidenbruch M. (2003). Reaction modes of a tetragermabutadiene: Cycloadditions versus Ge–Ge bond cleavages. Organometallics.

[53] Sasamori T., Sugahara T., Agou T., Sugamata K., Guo J.-D., Nagase S., Tokitoh N. (2015). Reaction of a diaryldigermyne with ethylene. Chem. Sci..

[54] Schäfer H., Saak W., Weidenbruch M. (2000). Hexaaryltetragermabuta-1,3-diene: A molecule with conjugated Ge–Ge double bonds. Angew. Chem. Int. Ed..

[55] Olmstead M.M., Pu L., Simons R.S., Power P.P. (1997). Reduction of Ge(Cl)C_6_H_3_mes_2_-2,6 to give the cyclotrigermenyl radical (GeC_6_H_3_mes_2_-2,6)_3_∙ and the trigermenyl anion salt K(GeC_6_H_3_mes_2_-2,6)_3_. Chem. Commun..

[56] Lee V.Y., Ito Y., Gapurenko O.A., Minyaev R.M., Gornitzka H., Sekiguchi A. (2020). From a (silatrigerma)cyclobutenylium ion to a (silatrigerma)cyclobutenyl radical and back. J. Am. Chem. Soc..

[57] Ramaker G., Schäfer A., Saak W., Weidenbruch M. (2003). One-pot synthesis of a tetragermabutadiene and its reactions with oxygen and sulfur. Organometallics.

[58] Hlina J., Baumgartner J., Marschner C., Albers L., Müller T., Jouikov V.V. (2014). Formation and properties of a bicyclic silylated digermene. Chem. Eur. J..

[59] Meyer H., Baum G., Massa W., Berndt A. (1987). Stable germaethenes. Angew. Chem., Int. Ed. Engl..

[60] Couret C., Escudié J., Satgé J., Lazraq M. (1987). The first stable germene: A compound with a germanium–carbon double bond. J. Am. Chem. Soc..

[61] Lazraq M., Escudié J., Couret C., Satgé J., Dräger M., Dammel R. (1988). (Mesityl)_2_Ge(fluorenylidene)–stabilization of a Ge–C double bond by charge transfer into an aromatic system. Angew. Chem. Int. Ed. Engl..

[62] Lazraq M., Couret C., Escudié J., Satgé J., Soufiaoui M. (1991). New stable germenes. Polyhedron.

[63] Anselme G., Escudié J., Couret C., Satgé J. (1991). Difluorenyl- and tert-butylfluorenyl(fluorenylidene)germenes: Synthesis, stabilization and first aspects of their reactivity. J. Organomet. Chem..

[64] Chaubon M.-A., Escudié J., Ranaivonjatovo H., Satgé J. (1996). Halogenogermenes: Evidence for the formation of the chloro- or fluoro-germenes (Me_5_C_5_)(X)Ge–CR_2_ (CR_2_ = fluorenylidene). J. Chem. Soc. Dalton Trans..

[65] Couret C., Escudié J., Delpon-Lacaze G., Satgé J. (1992). Dimesitylneopentylgermene, a new stable germene. Organometallics.

[66] Stürmann M., Saak W., Weidenbruch M., Berndt A., Scheschkewitz D. (1999). Molecular structures of new compounds with Ge=C and Sn=C double bonds. Heteroatom Chem..

[67] Meiners F., Saak W., Weidenbruch M. (2001). Reaction of a diarylgermylene with a phosphaalkyne: Formation of a germadiphosphacyclobutene with an exocyclic C=Ge double bond. Chem. Commun..

[68] Meiners F., Saak W., Weidenbruch M. (2000). Acetylene-linked bis(germaethenes): The first molecules with conjugated germanium–carbon double bonds. Organometallics.

[69] Meiners F., Haase D., Koch R., Saak W., Weidenbruch M. (2002). Cycloaddition reactions of an acetylene-linked bis(germaethene). Organometallics.

[70] Tokitoh N., Kishikawa K., Okazaki R. (1995). Synthesis and structure of the first germaketenedithioacetal. J. Chem. Soc. Chem. Commun..

[71] Lee V.Y., Ichinohe M., Sekiguchi A. (2002). 2,4-Disila-1-germatricyclo[2.1.0.0^2,5^]pentane: A new type of cage compound of group 14 elements with an extremely long Ge–C bridge bond and an “umbrella”-type configuration of a Ge atom. J. Am. Chem. Soc..

[72] Haas M., Leypold M., Schnalzer D., Torvisco A., Stueger H. (2015). Stable germenolates and germenes with exocyclic structures. Organometallics.

[73] Baines K.M., Cooke J.A. (1992). Tetramesitylgermasilene: The first relatively stable germasilene and its rearrangement to a silylgermylene. Organometallics.

[74] Ichinohe M., Arai Y., Sekiguchi A., Takagi N., Nagase S. (2001). A new approach to the synthesis of unsymmetrical disilenes and germasilene: Unusual ^29^Si NMR chemical shifts and regiospecific methanol addition. Organometallics.

[75] Sekiguchi A., Izumi R., Ihara S., Ichinohe M., Lee V.Y. (2002). The first isolable 1,1-dilithiogermane and its unusual dimeric structure—An effective reagent for the preparation of double-bonded derivatives of group 14 elements. Angew. Chem. Int. Ed..

[76] Igarashi M., Ichinohe M., Sekiguchi A. (2008). A stable silagermene (*^t^*Bu_3_Si)_2_Si=Ge(Mes)_2_ (Mes = 2,4,6-trimethylphenyl): Synthesis, X-ray crystal structure, and thermal isomerization. Heteroatom Chem..

[77] Al-Rafia S.M.I., Malcolm A.C., McDonald R., Ferguson M.J., Rivard E. (2011). Trapping the parent inorganic ethylenes H_2_SiGeH_2_ and H_2_SiSnH_2_ in the form of stable adducts at ambient temperature. Angew. Chem. Int. Ed..

[78] Ghadwal R.S., Roesky H.W., Merkel S., Henn J., Stalke D. (2009). Lewis base stabilized dichlorosilylene. Angew. Chem. Int. Ed..

[79] Majhi P.K., Huch V., Scheschkewitz D. (2021). A mixed heavier Si=Ge analogue of a Vinyl Anion. Angew. Chem. Int. Ed..

[80] Chaubon M.-A., Escudié J., Ranaivonjatovo H., Satgé J. (1996). First characterization of a compound with a tin-germanium double bond: The dimesityl(diisitylstanna)germene (Is)_2_Sn=Ge(Mes)_2_. Chem. Commun..

[81] Schäfer A., Saak W., Weidenbruch M. (2003). Tetraarylstannagermene: A molecule with a Ge=Sn double bond. Organometallics.

[82] Sekiguchi A., Izumi R., Lee V.Y., Ichinohe M. (2003). R_2_Ge=SnR′_2_ and RR′Ge=SnRR′ (R = SiMe*^t^*Bu_2_, R′ = 2,4,6-*^i^*Pr_3_C_6_H_2_): The new stable germastannenes. Organometallics.

[83] Lee V.Y., Takanashi K., Nakamoto M., Sekiguchi A. (2004). ^3^Δ-1,2,3,4-Disilagermastannetene: The first cyclic germastannene. Russ. Chem. Bull. Int. Ed..

[84] Stender M., Phillips A.D., Wright R.J., Power P.P. (2002). Synthesis and characterization of a digermanium analogue of an alkyne. Angew. Chem. Int. Ed..

[85] Stender M., Phillips A.D., Power P.P. (2002). Formation of [Ar*Ge{CH_2_C(Me)C(Me)CH_2_}CH_2_C(Me)=]_2_ (Ar* = C_6_H_3_-2,6-Trip_2_; Trip = C_6_H_2_-2,4,6-*i*-Pr_3_) via reaction of Ar*GeGeAr* with 2,3-dimethyl-1,3-butadiene: Evidence for the existence of a germanium analogue of an alkyne. Chem. Commun..

[86] Pu L., Phillips A.D., Richards A.F., Stender M., Simons R.S., Olmstead M.M., Power P.P. (2003). Germanium and tin analogues of alkynes and their reduction products. J. Am. Chem. Soc..

[87] Peng Y., Fischer R.C., Merrill W.A., Fischer J., Pu L., Ellis B.D., Fettinger J.C., Herber R.H., Power P.P. (2010). Substituent effects in ditetrel alkyne analogues: Multiple vs. single bonded isomers. Chem. Sci..

[88] Sugiyama Y., Sasamori T., Hosoi Y., Furukawa Y., Takagi N., Nagase S., Tokitoh N. (2006). Synthesis and properties of a new kinetically stabilized digermyne: New insights for a germanium analogue of an alkyne. J. Am. Chem. Soc..

[89] Wu P.-C., Su M.-D. (2011). Theoretical design for germaacetylene (RC≡GeR′): A new target for synthesis. Dalton Trans..

[90] Bibal C., Mazières S., Gornitzka H., Couret C. (2001). A route to a germanium–carbon triple bond: First chemical evidence for a germyne. Angew. Chem. Int. Ed..

[91] Bonnefille E., Mazières S., Saffon N., Couret C. (2009). Reactivity of a germa-alkyne: Evidence for a germanone intermediate in the hydrolysis and alcoholysis processes. J. Organomet. Chem..

[92] Berthe J., Garcia J.M., Ocando E., Kato T., Saffon-Merceron N., De Cózar A., Cossío F.P., Baceiredo A. (2011). Synthesis and reactivity of a phosphine-stabilized monogermanium analogue of alkynes. J. Am. Chem. Soc..

[93] Schäfer A., Weidenbruch M., Müller T., Pravinkumar K., Becker J.Y. (2009). Electrochemical properties of a disiliene, a tetrasila-1,3-butadiene, and their germanium analogues. Chem. Eur. J..

[94] Suzuki K., Matsuo T., Hashizume D., Fueno H., Tanaka K., Tamao K. (2011). A planar rhombic charge-separated tetrasilacyclobutadiene. Science.

[95] Suzuki K., Numata Y., Fujita N., Hayakawa N., Tanikawa T., Hashizume D., Tamao K., Fueno H., Tanaka K., Matsuo T. (2018). A stable free tetragermacyclobutadiene incorporating fused-ring bulky EMind groups. Chem. Commun..

[96] Lee V.Y., Ichinohe M., Sekiguchi A. (2000). The first metalladiene of group 14 elements with a silole-type structure with Si=Ge and C=C double bonds. J. Am. Chem. Soc..

[97] Lee V.Y., Ichinohe M., Sekiguchi A. (2001). Reaction of disilagermirenes with phenylacetylene: From a germasilene –Ge=Si– to a metalladiene of the type –Si=Ge–C=C–. J. Organomet. Chem..

[98] Lee V.Y., Kato R., Aoki S., Sekiguchi A. (2011). 1,3-Disila-3-germacyclopentadienes: Cyclopentadiene analogs based on heavier group 14 elements. Russ. Chem. Bull., Int. Ed..

[99] Cui C., Olmstead M.M., Power P.P. (2004). Reaction of Ar′GeGeAr′ (Ar′ = C_6_H_3_-2,6-Dipp_2_, Dipp = C_6_H_3_-2,6-*i*Pr_2_) toward alkynes: Isolation of a stable digermacyclobutadiene. J. Am. Chem. Soc..

[100] Cui C., Olmstead M.M., Fettinger J.C., Spikes G.H., Power P.P. (2005). Reactions of the heavier alkyne analogues Ar′GeGeAr′ (Ar′ = C_6_H_3_-2,6-(C_6_H_3_-2,6-Pr*^i^*_2_)_2_; E = Ge, Sn) with unsaturated molecules: Probing the character of the EE multiple bonds. J. Am. Chem. Soc..

[101] Sugahara T., Guo J.-D., Sasamori T., Karatsu Y., Furukawa Y., Ferao A.E., Nagase S., Tokitoh N. (2016). Reaction of a stable digermyne with acetylenes: Synthesis of a 1,2-digermabenzene and a 1,4-digermabarrelene. Bull. Chem. Soc. Jpn..

[102] Sugahara T., Sasamori T., Tokitoh N. (2018). 2,5-Digermaselenophenes: Germanium analogues of selenophenes. J. Am. Chem. Soc..

[103] Ishida S., Iwamoto T., Kabuto C., Kira M. (2003). A stable silicon-based allene analogue with a formally *sp*-hybridized silicon atom. Nature.

[104] Iwamoto T., Masuda H., Kabuto C., Kira M. (2005). Trigermaallene and 1,3-digermasilaallene. Organometallics.

[105] Iwamoto T., Abe T., Kabuto C., Kira M. (2005). A missing allene of heavy group 14 elements: 2-germadisilaallene. Chem. Commun..

[106] Sugahara T., Sasamori T., Tokitoh N. (2017). Highly bent 1,3-digerma-2-silaallene. Angew. Chem. Int. Ed..

[107] Eichler B.E., Powell D.R., West R. (1998). Synthesis and structure of a 1-germapropadiene. Organometallics.

[108] Tokitoh N., Kishikawa K., Okazaki R. (1998). Synthesis and reactions of the first stable 1-germaallene. Chem. Lett..

[109] Rit A., Campos J., Niu H., Aldridge S. (2016). A stable heavier group 14 analogue of vinylidene. Nature Chem..

[110] Krebs K.M., Hanselmann D., Schubert H., Wurst K., Scheele M., Wesemann L. (2019). Phosphine-stabilized digermavinylidene. J. Am. Chem. Soc..

[111] Jana A., Huch V., Scheschkewitz D. (2013). NHC-stabilized silagermenylidene: A heavier analogue of vinylidene. Angew. Chem. Int. Ed..

[112] Jana A., Majumdar M., Huch V., Zimmer M., Scheschkewitz D. (2014). NHC-coordinated silagermenylidene functionalized in allylic position and its behaviour as a ligand. Dalton Trans..

[113] Wang Y., Xie Y., Wei P., King R.B., Schaefer H.F., Schleyer P., von R., Robinson G.H. (2008). A stable silicon(0) compound with a Si=Si double bond. Science.

[114] Sidiropoulos A., Jones C., Stasch A., Klein S., Frenking G. (2009). *N*-heterocyclic carbene stabilized digermanium(0). Angew. Chem. Int. Ed..

[115] Jones C., Sidiropoulos A., Holzmann N., Frenking G., Stasch A. (2012). An *N*-heterocyclic carbene adduct of diatomic tin: Sn=Sn. Chem. Commun..

[116] Shan Y.-L., Yim W.-L., So C.-W. (2014). An *N*-heterocyclic silylene-stabilized digermanium(0) complex. Angew. Chem. Int. Ed..

[117] Sugahara T., Guo J.-D., Sasamori T., Nagase S., Tokitoh N. (2018). Regioselective cyclotrimerization of terminal alkynes using a digermyne. Angew. Chem. Int. Ed..

